# Robotont 3–an accessible 3D-printable ROS-supported open-source mobile robot for education and research

**DOI:** 10.3389/frobt.2024.1406645

**Published:** 2024-07-10

**Authors:** Eva Mõtshärg, Veiko Vunder, Renno Raudmäe, Marko Muro, Ingvar Drikkit, Leonid Tšigrinski, Raimo Köidam, Alvo Aabloo, Karl Kruusamäe

**Affiliations:** Intelligent Materials and Systems Lab, Institute of Technology, University of Tartu, Tartu, Estonia

**Keywords:** open-source hardware, educational robotics, 3D-printing, PCB design, robot design, modular hardware, Robot Operating System (ROS), citizen manufacturing

## Abstract

Educational robots offer a platform for training aspiring engineers and building trust in technology that is envisioned to shape how we work and live. In education, accessibility and modularity are significant in the choice of such a technological platform. In order to foster continuous development of the robots as well as to improve student engagement in the design and fabrication process, safe production methods with low accessibility barriers should be chosen. In this paper, we present Robotont 3, an open-source mobile robot that leverages Fused Deposition Modeling (FDM) 3D-printing for manufacturing the chassis and a single dedicated system board that can be ordered from online printed circuit board (PCB) assembly services. To promote accessibility, the project follows open hardware practices, such as design transparency, permissive licensing, accessibility in manufacturing methods, and comprehensive documentation. Semantic Versioning was incorporated to improve maintainability in development. Compared to the earlier versions, Robotont 3 maintains all the technical capabilities, while featuring an improved hardware setup to enhance the ease of fabrication and assembly, and modularity. The improvements increase the accessibility, scalability and flexibility of the platform in an educational setting.

## 1 Introduction

Educational robots offer a platform for training aspiring engineers and, on a broader scale, building trust in technology that is envisioned to shape how we work and live. However, as with many emergent technologies, barriers related to the accessibility of educational robots persist potentially due to the early development stage or high cost of the available solutions (Reich-Stiebert and Eyssel, 2016).

The integration of open-source hardware in educational robotics has been proposed as a potential solution (Heradio et al., 2018; Patel et al., 2023) as it holds a promise of accessible, customisable and transparent development. However, being open-source does not guarantee universal accessibility, as the fabrication might be costly or call for techniques normally beyond the reach of educators or self-guided learners.

In recent years, 3D-printing has become a viable alternative, poised to democratise open-source hardware and improve the accessibility of open-source educational robotics by mitigating barriers associated with traditional fabrication methods (Lapeyre et al., 2014; Voellmy and Ehrhardt, 2020). Simultaneously, developing custom printed circuit boards (PCBs) has grown increasingly accessible and less cost-prohibitive (Hodges and Fraser, 2022). A combination of these technologies could thus potentially be leveraged to enhance the accessibility and affordability of open-source educational robotics.

**FIGURE 1:**
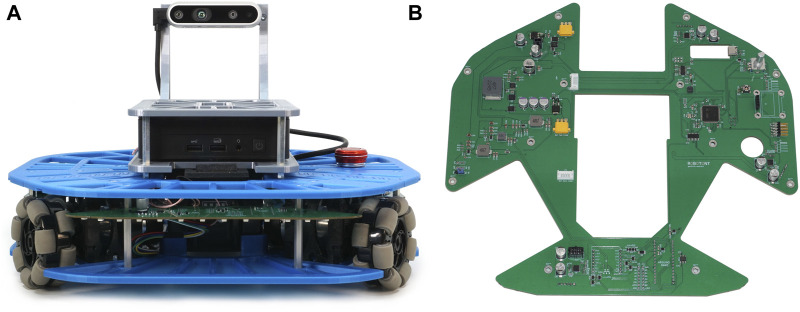
**(A)** Front view of Robotont chassis; **(B)** Top view of Robotont PCB.

This paper introduces a 3D-printed open-source omnidirectional mobile robot Robotont 3 designed as a dedicated platform for both education and research. Building on our prior work (Raudmäe et al., 2023), we have enhanced the robot’s design by switching to a fully 3D-printed chassis (Figure 1A) and consolidating the electronics into a single system board (Figure 1B) that can be readily ordered from an online PCB manufacturing service. These improvements make it easier to manufacture and assemble the robot by someone who does not have access to CNC-milling and tools for electronics assembly.

The 3D-printed mechanical components of the third generation Robotont are optimised for print bed dimensions of 250 × 210 mm, ensuring compatibility with the majority of commonly used consumer-level printers. By leveraging the inherent advantages of additive manufacturing, we have achieved a 32% reduction in the chassis mass (11% reduction in total robot mass). The introduction of the consolidated PCB has significantly improved assembly time and durability of connections. We have replaced the volatile lithium polymer (LiPo) batteries of the previous version with an off-the-shelf 18 V Makita lithium ion (Li-Ion) battery, which is safe to use and charge due to integrated control circuits, and widely accessible globally. The new iteration of Robotont incorporates modular design in order to boost the ease of production and modification. Collectively, these advancements markedly enhance Robotont’s accessibility for educators, self-guided learners and robotics researchers.

Robotont 2 was intended to bridge the gap between accessible but computationally more limited educational robots and non-accessible but computationally capable industrial robots, offering a reasonably priced platform for teaching industry-relevant ROS skills in higher education without the need for industry-priced platforms (Raudmäe et al., 2023). Robotont 3 continues with this motivation, with the addition of bringing the manufacturing of the robot closer to target audiences. While several educational robots with open-source hardware have been published in recent years, such as WitBot (Miller-Klugman et al., 2022) and OpenScout (Carter et al., 2023), they often do not have ROS capabilities nor the compute power required for running state-of-the-art algorithms.

Similarly to its predecessor and other ROS-supported mobile robots such as the Clearpath Turtlebot 4 (Clearpath Robotics, Inc., 2022b), Thymio (Mondada et al., 2017), ExoMy (Voellmy and Ehrhardt, 2020) and ROMR (Nwankwo et al., 2023), Robotont 3 has full ROS support and preconfigured key capabilities associated with mobile robots, e.g., teleoperation, 2D simultaneous localization and mapping, autonomous navigation, 3D-mapping, and tracking of fiducial markers. All ROS features available on Robotont 2 (Raudmäe et al., 2023) are also fully functional on Robotont 3, and as such, the position of Robotont 3 in the ROS ecosystem remains unchanged. The contribution of this article is to describe the updated physical platform that is fully compatible with the high-level software stack of its predecessor.

## 2 Hardware description

### 2.1 About Robotont

**FIGURE 2:**
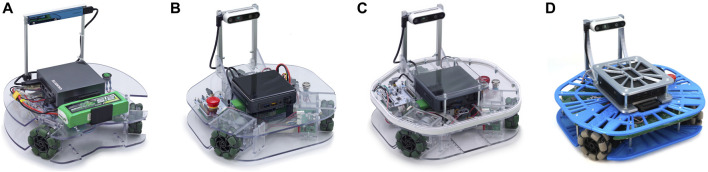
Evolution of the Robotont robot. Generations from left to right: gen 1 **(A)**, gen 2 **(B)**, gen 2.1 **(C)**, gen 3.0 **(D)**.

This paper describes the design of the third generation of the Robotont robot (Figure 2D). Robotont (Figure 2) is an omnidirectional wheeled platform with a flat shape and low bottom (outer dimensions 326 × 300 × 225 mm for width, length and height respectively). The robot has three omniwheels, an onboard Intel^®^ NUC computer and an Intel^®^ RealSense™ depth camera. The open-source specification and resources of Robotont are listed in Table 1. Before Robotont 3, there have been three earlier versions (Figures 2A–C).

**TABLE 1:** Hardware specifications.

Hardware name	Robotont 3
3D-printing method	Fused Deposition Modeling (FDM)
Open-source license	CERN-OHL-P-2.0 for the hardware Apache 2.0 for the software
Cost of hardware	1500 EUR
Source file repository	doi.org/10.5281/zenodo.12205274
	github.com/robotont/robotont-frobt-2024-replication-package
	github.com/robotont
Bill of Materials	Supplementary Material S1
Assembly instructions	Supplementary Material S2

Robotont 3 has numerous advancements compared to the previous version [Figure 2C, Raudmäe et al. (2023)]. Of these, the most significant are the new 3D-printed chassis and the comprehensive PCB that joins together previously separate electronics. The following subsections will describe both of these developments in detail.

Additionally, with the introduction of Robotont 3, we adopt Semantic Versioning (SemVer) (Preston-Werner, 2013) to keep track of changes and make it easier to distinguish between the different iterations of individual components. The versioning system offers a robust way of ensuring compatibility between multiple developers’ work. It also makes it easier for the users to validate if the assembly instructions and other materials are compatible with their version of Robotont.

### 2.2 3D-printed chassis

**FIGURE 3:**
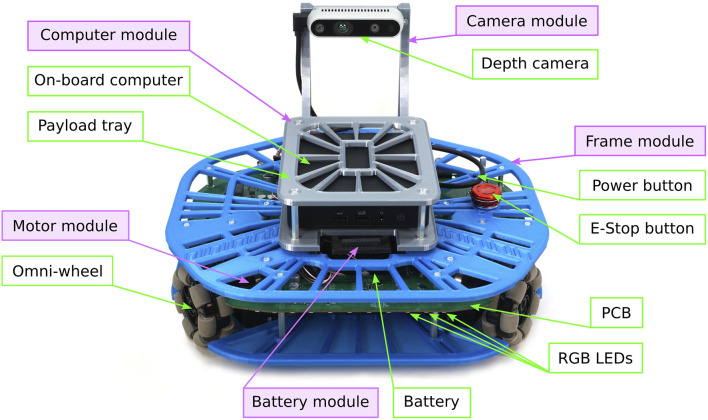
Fully assembled Robotont robot. Green clear boxes denote some major components of the robot; purple shaded boxes denote the 3D-printed chassis modules.

**FIGURE 4:**
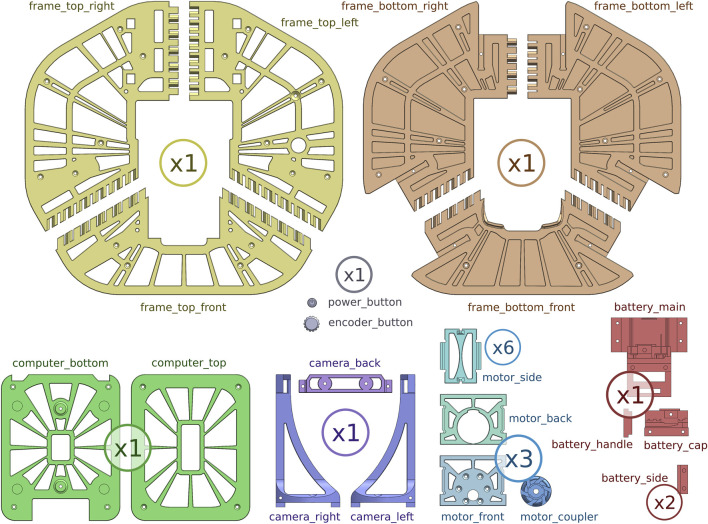
Mechanical components that need to be 3D-printed to assemble Robotont, to scale. Numbers denote how many of each component is needed.

**FIGURE 5:**
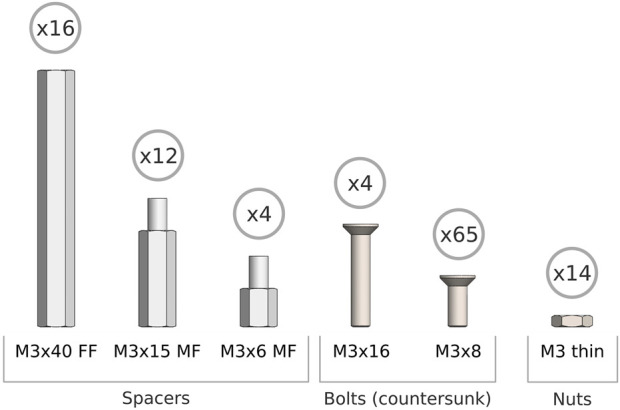
Fastenings needed for assembly of the 3D-printed chassis.

**FIGURE 6:**
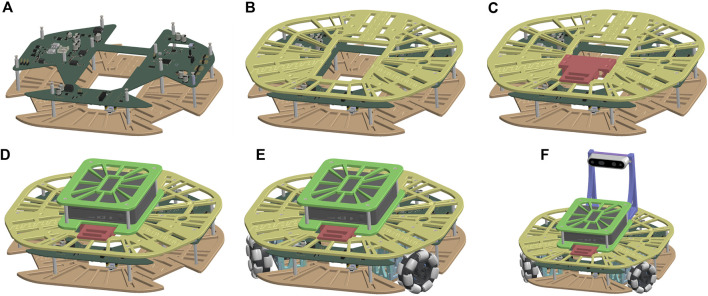
Simplified assembly steps: **(A)** PCB is attached to bottom part of frame module, **(B)** frame module is completed, **(C)** battery module is added, **(D)** computer module is added, **(E)** motor modules are added, **(F)** camera module is added.

The chassis of Robotont 3 consists of five types of modules (Figure 3): frame module, computer module, camera module, battery module and motor modules. Every module has a core component around which it is built, such as the RealSense™ depth camera for the camera module. The modular design makes it easy to swap components and gives a greater freedom in creating varying colour schemes for the chassis. All the 3D-printed mechanical components, including their names and required amounts, are shown in Figure 4, and the fastenings are shown in Figure 5. Figure 6 shows an overview of how the modules are assembled into a complete Robotont robot. To enable replication, all model files are available as both SolidWorks and STEP files in Zenodo and the Robotont mechanics repository (Table 1).

**FIGURE 7:**
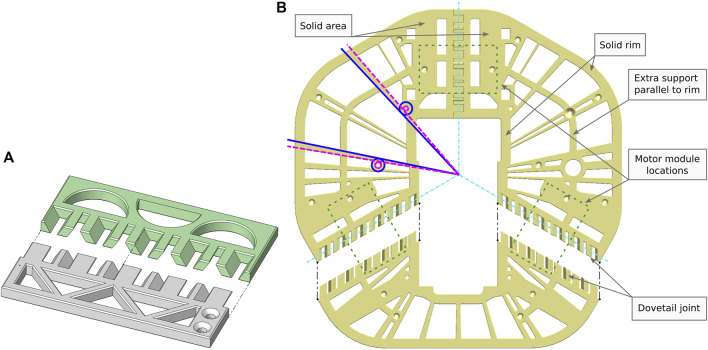
**(A)** 3D-printer calibration pieces showcasing the dovetail joint used in Robotont frame module. **(B)** Example of the radial skeleton rays design element in the frame module. The light blue dash-dot lines depict how the outer edges of the three parts converge in the centre of the robot’s body. The dashed pink circles show examples of functional holes and dashed pink lines their tangents; solid blue circles show examples of solid rims around the functional holes and solid blue lines show their tangents accordingly. The black dashed lines show the direction of connection of these particular parts. The extra support parallel to rim improves rigidity by preventing excessively large open areas. The green dotted lines show the locations of the three motor modules.

All 3D-printed chassis components incorporate a lattice-like structure. The lattice consists of solid outer edges and an internal skeleton that is based on radial rays (Figure 7B) that are formed by the tangents of structurally relevant holes and the solid rims around them. The locations of skeleton rays are chosen so that every structurally relevant component, such as spacer attachment points, would be secured to the rest of the structure from at least two sides. Additionally, every such component adds skeleton rays that extend to the edge of the part. In places with too few such components, extra rays are added at approximately even intervals. Such design is meant to provide rigidity while keeping the parts lightweight.

3D-printed objects consist of solid perimeters (the visible part) and non-solid infill (the internal part with air gaps). Perimeters, being solid material, offer more rigidity than infill (Mazlan et al., 2023). Since the Robotont chassis parts are 3D-printed, having a lot of perimeters in the design is preferable. Another advantage of the directional skeleton is that it ensures that when the robot collides with obstacles in its environment, one of the rays is always parallel to the direction of the collision, thus potentially preventing deformations.

The overall dimensions of Robotont (326 × 300 mm) exceed the print areas of most popular consumer-level FDM printers, such as Bambu Lab P1P (print area 256 × 256 mm), Prusa MK3S (print area 250 × 210 mm), Creality Ender 5 (print area 220 × 220 mm) and Anycubic i3 Mega (print area 210 × 210 mm). To make the chassis printable in consumer-level printers, the two large flat plates of the frame module are divided into three separate parts (Figures 4, 7B). The locations for connecting them were chosen with three considerations in mind: 1) keeping the number of parts low, 2) while still making sure they all fit within the print area, and 3) ensuring mechanical sturdiness.

For mechanical sturdiness the connections were placed above and below the motor modules, aligning with the middle axis of the motor modules (Figure 7B). This way the connections between the frame and motor modules also reinforce the connections between the individual parts of the flat plates.

To connect the parts of the flat plates, we used a mechanical fastening-free joint inspired by dovetail joints (Figure 7A). The dovetail joint is frequently used in carpentry and is mechanically resilient thanks to slanted faces and trapezoidal shapes (Wengang Hu et al., 2023). In 3D-printing, an added benefit of the dovetail joint is that it requires no support structures. Printing the joints without support structures is crucial to achieve smooth surfaces and a consistent fit.

**FIGURE 8:**
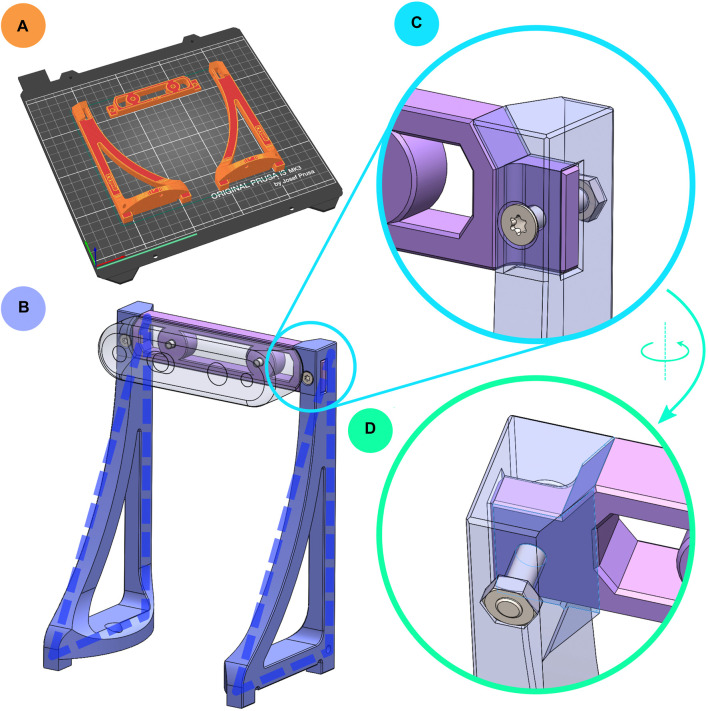
**(A)** Printing orientation of camera module components. **(B)** Camera module, with triangular sides highlighted with dashed blue lines. **(C)** Close-up view of the camera module’s pinned mortise and tenon joint, shown semi-transparently. **(D)** View from behind the joint; the surfaces highlighted in purple provide additional support to the joint when the robot is lifted.

The protruding camera mount (Figure 8) is often the easiest to grab when lifting the robot. To improve the ease of handling Robotont, the camera module is designed to double as a handle, instead of cautioning the users not to use it for lifting the robot. For the camera mount to safely carry Robotont’s entire mass, the sides of the camera module are triangular (Figure 8B) to prevent them from breaking when the robot is lifted. Furthermore, in 3D-printing, a regular failure mode stems from faults in layer adhesion, while the layers themselves are relatively resilient (Bellini and Güçeri, 2003; Gurrala and Regalla, 2014; Penumakala et al., 2020). To maximise layer surface areas and minimise the risk of breaking, the camera module comprises three components that are all printed flat (Figure 8A). The camera is connected to a horizontal back-piece, which is secured to the two triangular sides by a pinned mortise and tenon joint on both ends (Figure 8C). Additional slanted support surfaces help to further distribute some of the load from the joints (Figure 8D).

All components of the chassis are designed to be printed without supports to minimise material and time consumption. The fit of interlocking parts is chosen as a balance between sufficiently tight and achievable in varying printing conditions. To aid assembly and to counteract printer temperature control differences, 0.5 mm 45-degree chamfers are added to all edges. This reduces the risk of elephant foot–a common 3D-printing failure mode where the bottom of a part expands outward due to a combination of too high temperature and the weight of the printed part. In Robotont, especially in the frame module, it is paramount to avoid elephant foot in order to ensure that the parts can fit together. To verify that the printing settings are suitable for the given 3D-printer, a calibration piece (Figure 7A) can be printed. If the two-halves fit together easily, it is more likely that the robot parts will fit as well. If the fit is bad, the surfaces are not smooth or if elephant foot is present, the printing settings can be tuned without printing out the full robot.

### 2.3 Electronics

A consolidated approach is adopted in the third generation of the Robotont platform, integrating all electronic components seamlessly onto a single PCB. This design substantially differs from the previous concept, where various submodules were connected using wires and connectors (Raudmäe et al., 2023). The onboard establishment of connections through PCB traces mitigates the risk of connection failures between the modules and streamlines the assembly process, resulting in cost savings.

#### 2.3.1 Architectural overview

**FIGURE 9:**
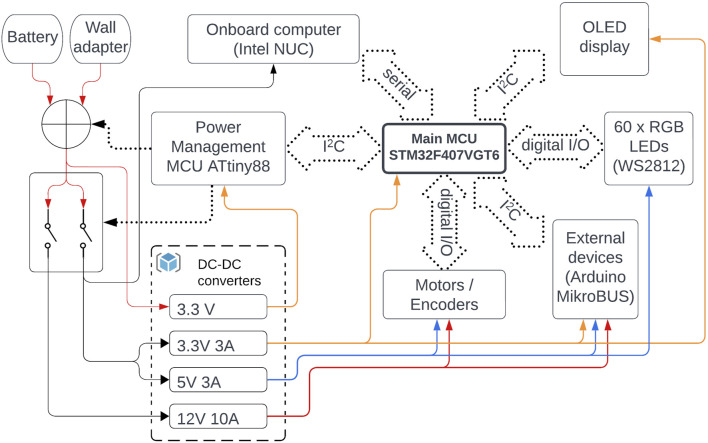
Architectural overview of Robotont 3 electronics. Solid lines represent power paths. Dotted contours and lines represent data communication and control signals.

At the highest level, Robotont features a 13th generation Intel NUC computer, which is powered by Ubuntu Linux 22.04 and equipped with ROS (Robot Operating System) software. It orchestrates the entire robot and communicates to the mainboard via a serial interface (Figure 9). The mainboard operates with two microcontroller units (MCUs): an ATtiny88 (Atmel) for low-level power management and an STM32F407VGT6 (STMicroelectronics) for central coordination of communication.

Operational instructions received from the robot’s onboard computer are processed and executed on the main MCU, which integrates other subsystems and peripherals, including motors, encoders, and a display. The instructions are sent using a custom communication protocol implementing the following Robotont packet format “cc:arg1:arg2:….:argN∖r∖n”. The packet starts with a two-character operational command that is followed by its arguments, with a colon character serving as the separator. Packets are terminated by linefeed, newline, or carriage-return characters. The current command set allows setting robot velocities in either robot coordinates (RS) or for each wheel individually (MS). Furthermore, the DC command facilitates the direct configuration of the motor control signal’s duty cycle, thereby disabling the PID controller typically engaged in RS and MS modes. The main MCU uses OD command to transmit processed wheel odometry information back to the onboard computer. The following sections will discuss the key subsystems of the mainboard and design considerations in more detail.

#### 2.3.2 Power management

The robot can be powered either from an 18 V Makita Li-Ion battery or an external power supply (recommended input voltage range 14–24 V). Both inputs use 3-pin AMASS MR30PW connectors with additional 6-pin data connectors. Relaying all battery pins becomes of importance when envisioning future dock-based charging capabilities. Furthermore, the hardware enables monitoring of one of the data pins, where the voltage correlates to the charging current. This feedback is essential to verify whether the docking process has succeeded or if the charging is complete.

The mainboard hosts four DC-DC voltage converters: 12 V, 5 V, and two 3.3 V units (Figure 9). One of the 3.3 V converters is set in a constant-on configuration to power the ATtiny88 power management microcontroller. Besides power path switching, this controller is also responsible for other low-level tasks, such as monitoring input voltages and currents, monitoring the positions of the power-on and stop switches, activating a piezoelectric buzzer at a low battery level, and reporting the status information to the main MCU over the I^2^C bus.

#### 2.3.3 Motors

The robot’s three omnidirectional wheels are operated by the 12 V DC motors of type 37Dx68L. Each motor has a 64 counts per revolution encoder attached to the end of the motor shaft and includes a gearbox with a reduction ratio of 19:1, which makes the wheels revolve in unloaded conditions up to 530 RPM (or 1.9 m/s linear speed perpendicular to the shaft axis). The DRV8874 (Texas Instruments) driver chips are employed to ensure precise control over the velocity and direction of the motor movements. Given that both the motor drivers and encoders operate on a 5 V logic level but the main MCU is running at 3.3 V, the signals to the driver pass a π 160U (2Pai Semi) digital isolator chip to ensure proper signal integrity and provide additional isolation from the 5 V and 12 V circuits.

#### 2.3.4 User interface

The third generation of Robotont comes with a minimalistic user interface consisting of a rotary encoder push button and a standard 128 × 64 OLED display, both of which are integrated into the PCB and connected directly to the main microcontroller. The interface enables fundamental interaction with the robot without the need for a network connection or the attachment of external peripherals such as a keyboard, mouse, or HDMI display. For example, one can display debug information and robot status, change operating modes, initiate shutdown sequence, etc.

#### 2.3.5 Additional device connectivity

Robotont’s capabilities can be expanded through a designated area on the front of the PCB. The area exposes a dedicated power connector with 12 V, 5 V, and 3.3 V supplies and sockets for Arduino Nano and MikroBUS devices supporting both I^2^C and SPI standards with selectable 3 V or 5 V logic levels. In addition to the Arduino Nano socket, all the leftover Arduino pins are exposed with generic pin headers. This configuration makes it possible for the user to interface miscellaneous devices with the Arduino and enables the implementation of an additional data processing step before needing to communicate with the mainboard MCU. The Arduino Nano’s integration on the Robotont PCB simplifies the pathway for entry-level users to conduct rapid testing and interface with a diverse range of actuators and sensors.

For communication, the Arduino, MikroBUS, and STM32 microcontroller all operate on a shared I^2^C interface that can be utilized to establish bi-directional communication between additional devices and higher-level software running on the onboard computer. While the hardware supports this communication, the exact communication protocols for additional devices have not yet been finalised.

#### 2.3.6 PCB layout considerations

**FIGURE 10:**
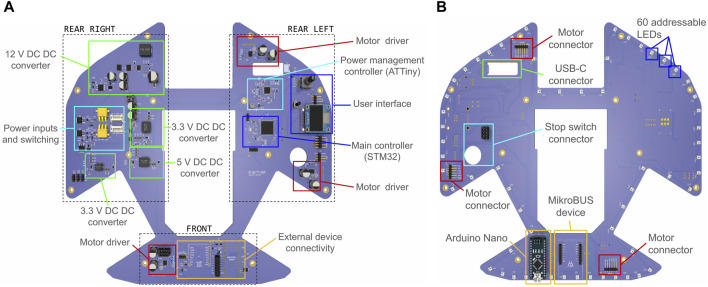
Robotont 3 PCB: **(A)** top side; **(B)** bottom side.

The PCB on which all electronic components are placed conforms to the robot’s outer perimeter. It contains wheel and battery cutouts, resulting in a shape with three distinct areas for placing components (Figure 10). The rear right area is dedicated to power management, the rear left area houses the main controllers and user interface elements, and the front area is for attaching additional devices. The layout also aims to keep additional wired connections to the components as short as possible, thus defining the locations of the motor drivers close to the wheel modules, for example.

The PCB has two layers. The top layer (Figure 10A) predominantly hosts most of the functional components, whereas the ports for connecting motors, a stop switch, and external devices are placed on the bottom layer (Figure 10B). Additionally, the outer edge of the bottom PCB layer is populated with 60 addressable RGB LEDs to provide downwards-facing visual feedback for users and enable the creation of light-based expressions.

### 2.4 Firmware and programming

Both microcontrollers integrated onto the PCB are accessible for programming via pin-header connectors placed near the user interface area. More specifically, a 2 × 4 pin SWD Debug port and a generic 2 × 3 pin ISP header facilitate the code upload and debugging for the main MCU and the power management MCU, respectively. A cost-effective programmer for the power management microcontroller involves employing an Arduino Nano with the Arduino as ISP firmware, which is a built-in example within the Arduino IDE. The programming of the main MCU is performed using the NUCLEO-L476RG (STMicroelectronics) development board that has an integrated ST-LINK/V2-1 debugger/programmer.

Unlike the power management MCU, the main MCU assumes more complex roles. It serves as a communication hub between the onboard computer, power management system, and external devices. Additionally, it monitors motor speeds via encoder pulse readings and employs PID controllers to regulate each motor’s speed independently. Furthermore, it manages the content displayed on the OLED display and controls the addressable LED pixels. The main MCU can receive commands from the onboard computer to address the LEDs individually, in segments, or to change the current lighting mode. In different lighting modes, the MCU controls the LEDs to create various lighting visuals.

**FIGURE 11:**
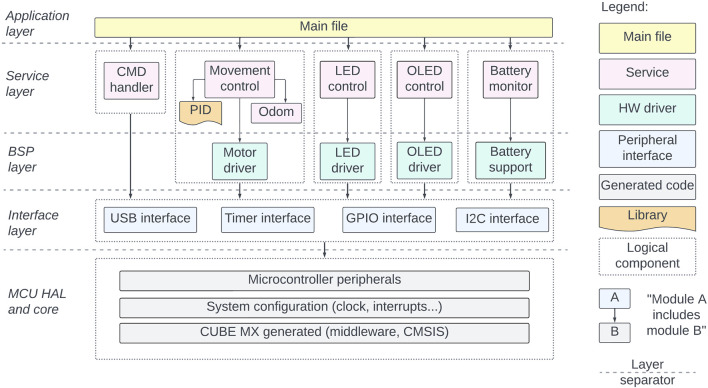
Robotont 3 firmware.

A robust architectural foundation and adherence to coding style guidelines are essential to integrate all the above-mentioned functionality into a single solution. To this end, we have structured the firmware implementation into five layers: Application, Service, BSP (Board Support Package), Interface, and MCU HAL (Hardware Abstraction Layer) and core (Figure 11). The Application layer governs interaction among services, while the Service layer handles autonomous tasks with non-blocking updates. The BSP layer contains hardware-specific firmware drivers, and the Interface layer simplifies communication with MCU peripherals. At the lowest level, MCU HAL and core components facilitate hardware interaction. Such progression of abstraction levels from the hardware-specific core toward the service and application layers provides a modular and convenient development framework, enabling seamless integration of potential future hardware modifications into the codebase. For maintaining clarity and consistency in the present and future developments, a more detailed architecture description and coding style guide with examples are provided in Zenodo and the Robotont firmware repository (Table 1).

## 3 Results and validation

### 3.1 Manufacturing of Robotont 3

**FIGURE 12:**
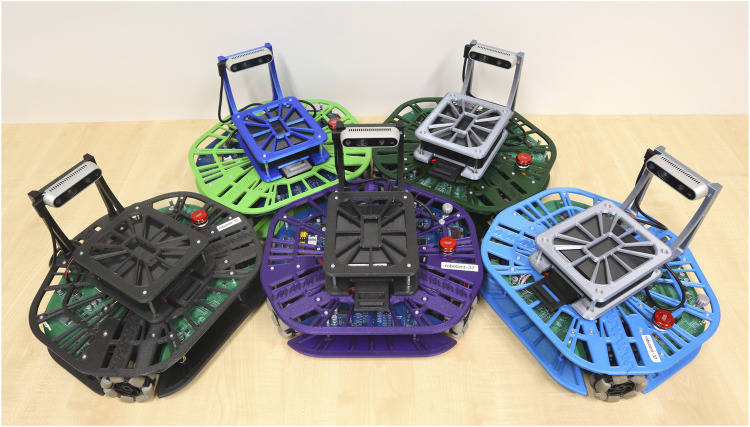
Different-coloured Robotont robots. The use of 3D-printing makes it possible to easily create different-coloured robots either for aesthetic or functional purposes.

We have produced several Robotont 3 robots in different colour combinations (Figure 12).

All components needed for the fabrication of Robotont 3 are listed in a comprehensive Bill of Materials (BOM), available in Supplementary Material S1. To support the replication of Robotont either in an educational or personal setting, thorough step-by-step assembly instructions are provided in Supplementary Material S2. A video example of a fully assembled and functional Robotont 3 is provided in Supplementary Material S3.

**FIGURE 13:**
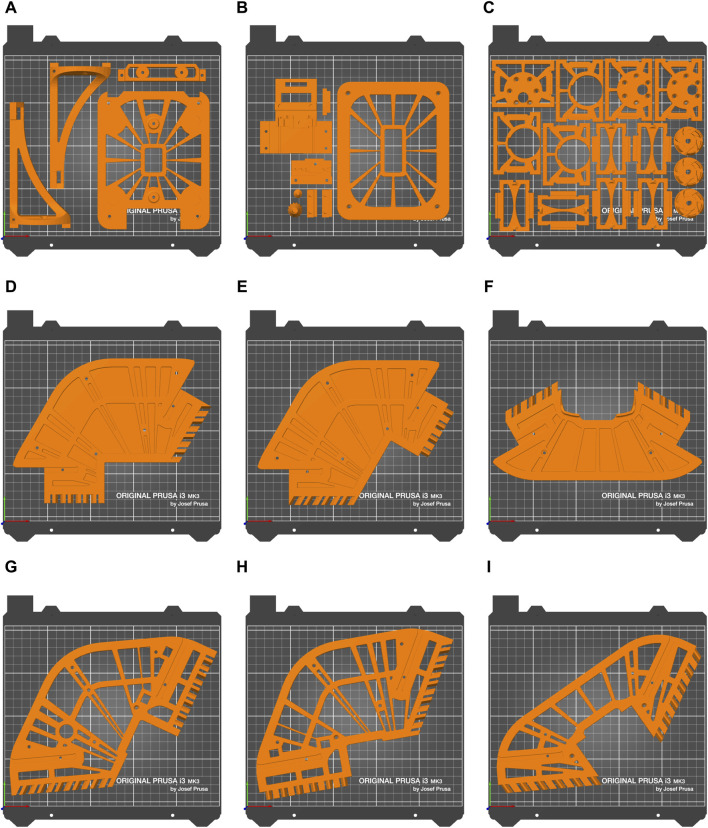
Printing batches of Robotont optimised to minimise human time consumption. Batch names and print times of every batch are as follows: **(A)** cam_comp_1 9 h 4 min **(B)** cam_comp_2 7 h 22 min **(C)** motor_all 13 h 40 min **(D)** frame_bottom_left 5 h 32 min **(E)** frame_bottom_right 5 h 33 min **(F)** frame_bottom_front 4 h 40 min **(G)** frame_top_left 5 h 39 min **(H)** frame_top_right 5 h 32 min **(I)** frame_top_front 4 h 44 min.

All 3D-printed parts were produced with Prusa MK3S printers. The main printer parameters that were tuned were infill percentage and perimeter count. Combinations of infill percentages (15%, 30%, 40%, 50%, 70%) and perimeter counts (3, 5) were compared empirically, settling on a combination of 40% rectilinear infill and 5 perimeters. All parts were printed with PLA, except motor module parts, which were printed with PETG to better withstand the heat generated by the motors. The total print time for the entire Robotont is 62 h. The robot can be printed in a minimum of nine batches (Figure 13). To enable replication, 3MF files of all print batches are available in Zenodo and the Robotont mechanics repository (Table 1).

The PCB was designed in KiCad software (KiCad, 2024). The manufacturing and assembly service of all SMD components was ordered from JLCPCB. For a convenient export of compatible manufacturing files, a JLCPCB Tools plugin was added to KiCAD. Components requiring through-hole technology were assembled manually in the University of Tartu facilities.

An overview of how Robotont 3 compares to the previous generation in technical aspects is shown in Table 2.

**TABLE 2:** Technical properties of Robotont 3 in comparison to Robotont 2.

Property	Robotont 3	Robotont 2
Dimensions (width x length x height mm)	326 × 300 × 225	326 × 300 × 240
Chassis production method	FDM 3D-printing	CNC-milling
Chassis material	PLA + PETG	Polycarbonate
Mass of chassis (kg)	0.95	1.4
Total mass (kg)	3.3	3.7
Number of chassis components (excl fasteners and buttons)	31	34
Number of PCB’s	1	6
Number of custom cables (number of wires)	4 (17)	10 (38)
Tentative cost of electronics (EUR)	200	150
Tentative cost of chassis (EUR)	30	200

### 3.2 Piloting in educational settings

Robotont 3 has been evaluated successfully in several educational settings. The most significant of these was a massive online open course (MOOC) in Estonia, which focused on teaching ROS on physical Robotont robots to uninitiated students over a remote web interface (Schumann et al., 2023; Krumins et al., 2024). The students could book time slots for using the robots, with the regular length being 85 min. Three Robotont 3 robots were piloted alongside four Robotont 2 in the second run of the MOOC, which was conducted over a 2-month period in the fall of 2023. In this educational setting Robotont 3 was found to offer a significant advantage over Robotont 2 in terms of both battery life and ease of battery replacement. No comprehensive analysis was made, but battery life enabled the students to continuously use the Robotont 3 over at least two consecutive 85-min slots, whereas the Robotont 2 required battery replacement after every slot. The battery location on Robotont 3 also proved to be much more conveniently accessible than that of Robotont 2. In total, the three Robotont 3 robots were in use by students for over 85 h each during the MOOC.

In a different educational context, portable kits containing a Robotont robot and all of its supporting devices and materials (such as batteries, charging station, handheld console for remote control, etc.) were developed and evaluated in teacher trainings with 37 participants (Raudmäe et al., 2024). The kits were compiled and the trainings were conducted in both Estonia and Austria. Robotont 3 was exclusively used in Estonia, while Robotont 2 was used in Austria. The battery was again highlighted as a safety concern in Robotont 2, further showcasing the marked improvement in the choice of power supply in Robotont 3.

Robotont 3 has already been used for furthering the robotics education of the students of University of Tartu through providing them with a platform for their student theses. In 2024, five Bachelor’s theses focused on developing new functionality for Robotont 3 were defended, with potential for several more in the future.

## 4 Discussion

### 4.1 Benefits of 3D-printing

In addition to 3D-printing being a safer and more accessible fabrication method than CNC-milling, the 3D-printed design offers four additional benefits over the CNC-milled chassis of the previous Robotont: 1) reduction in mass, 2) reduction in waiting time, 3) increase in ease of modification, 4) reduction in cost. All of these are explained in more detail in this subsection.

The chassis of Robotont 3 weighs *ca* 0.95 kg, of which 0.82 kg are the 3D-printed parts and the rest are the metal spacers. The chassis of the polycarbonate version weighs *ca* 1.4 kg. Through leveraging the benefits of additive manufacturing as opposed to subtractive, a weight reduction of 0.45 kg was achieved. This amounts to 32% of the entire chassis mass, which is significant in a mobile robot, boosting both the speed and the battery life.

With the combination of settings used in this work, the print time of the entire Robotont chassis is roughly 62 h. However, the actual human time consumption is mostly limited to starting the prints and removing finished parts from the printer, which we estimate to take around 3 h in total, with the entire chassis being printable in 9 prints (Figure 13) and estimating 20 min of human attention per print. Tentatively, CNC-milling the same parts with non-industrial machinery would take around 7 h of human time, of which 1 h comprises preparation and mill setup and 6 h is milling time when the operator would need to keep an eye on the mill.

The advantage of 3D-printing over CNC-milling in terms of operator time consumption becomes even more apparent when considering the ease of making modifications to the design. To replace a broken part or test out a new design, it simply needs to be printed. As the same set of printing settings can be used as for the previous part, slicing the modified part in slicer software takes virtually no time. This is an advantage over CNC-milling, where even if one had easy access to the mill, the toolpath would still need to be set up for the new geometry of the part, which is a manual process requiring expert knowledge.

The ease of modification is further supported by the increased modularity of the chassis, most notably in the frame module. The module consisting of six smaller parts instead of two large ones enables re-printing only one of them if a small change is needed. This saves both production time and material, as it is possible to replace just a third of the large flat area instead of the entire area. Both the choice of 3D-printing as a manufacturing method and the modularity of the parts contribute to the ease of adding new functionality in the future, hence supporting the continued development of Robotont.

In addition to streamlining development of new features, the 3D-printable design also enables the user to easily vary materials based on custom requirements. For example, if a more durable robot is needed, nothing in the design would need to be changed because all of the required modifications would happen in the slicer software. Different colour combinations are also easily achievable with 3D-printing, adding both aesthetic and functional opportunities. For example, with different-coloured robots it would be possible to detect them based on colour, which provides a learning opportunity in the context of computer vision.

For the average user, 3D-printing is a safer production method than CNC-milling, in which material is cut from a solid piece with a rapidly moving blade. 3D-printing also requires less specialised knowledge to use, and the acquisition costs of 3D-printers are much lower compared to CNC-mills. For these reasons, the 3D-printable chassis would be feasible to produce at school or at home, whereas crafting the previous version with a CNC-mill would be unrealistic in these settings. This introduces a difference in chassis cost. The CNC-milled version, which would need to be ordered from a manufacturer, entails both material and production costs, which amount to *ca* 200 EUR. The 3D-printed version entails only material cost, which is *ca* 25 EUR if using Prusament filaments, plus the cost of the metal spacers connecting the 3D-printed parts, which was around 7 EUR. Hence for the average user, the cost of Robotont’s chassis has been reduced roughly seven times.

### 4.2 Scalability

Even though the printing requires around 3 h of human input, the 62 h of printer time still keeps the printer occupied for that entire duration. For this reason, the scalability of our proposed method is hampered if many robots are needed in a short amount of time. The longest batch printing time is 13 h 40 min for motor module components (Figure 13C). If nine printers could be used simultaneously, this would be the limiting factor in printing time. After this time, all of the prints would be complete. Given an unlimited number of printers, the limiting factor would be the longest single part printing time, as all parts could then be printed individually. In this parallel printing scenario, all of the parts would be printed after 5 h 39 min, which is the printing time for frame_top_left (Figure 13G).

The consolidated PCB of Robotont 3 significantly enhances scalability and is better suited for mass production. Since the operator’s involvement in preparing cable harnesses and assembling connectors is minimized, approximately 1 h of human labor per robot is saved solely on wiring, compared to previous Robotont versions.

### 4.3 Software

Even though the firmware of Robotont 3 was completely redesigned as described in Section 2.4, the main ROS-based software system remains unchanged from the previous generation. Currently, the main software stack uses ROS 1 Noetic but ROS 2 Humble support is being actively developed and already available for several packages under dedicated branches on Robotont’s GitHub (Table 1). New developments with Robotont 3 will prioritise ROS 2, as is the prevalent development direction for other mobile robots (Clearpath Robotics, Inc, 2022a; Clearpath Robotics, Inc, 2022b; PAL Robotics, 2023).

The main ROS functionalities of Robotont 3 are teleoperation, 2D simultaneous localization and mapping, 3D-mapping, autonomous navigation, and tracking of fiducial markers. All these functionalities are present in the high-end research and industrial robots such as the Clearpath Jackal (Clearpath Robotics, Inc., 2022a) and PAL Robotics TIAGo (PAL Robotics, 2023) as well as in some of the more affordable alternatives such as Clearpath Turtlebot 4 (Clearpath Robotics, Inc., 2022b) and ROMR (Nwankwo et al., 2023). However, due to the increased compute capabilities stemming from the onboard Intel^®^ NUC, Robotont 3 might have a competitive edge in these operations in comparison to robots that instead use a Raspberry Pi or similar computer, which is often the case for the more affordable platforms.

### 4.4 Improved accessibility for education and research

The 3D-printable Robotont offers distinct advantages over the previous CNC-milled version due to its accessibility and affordability. Compared to CNC-mills, 3D-printers are safe to use even for a beginner. This makes 3D-printing suitable for students to try out customising the design without fear of injury. Iterating designs quickly and experimenting with various configurations can foster creativity and improve problem-solving skills. By customising, 3D-printing and assembling a Robotont at school, students can get hands-on experience about the manufacturing process, which promotes a deeper understanding of the field of robotics. Moreover, the relatively low cost of 3D-printers and materials makes them more feasible for educational institutions with limited budgets, ensuring broader access to robotics education.

For researchers, the modular design and ease of making modifications can offer a flexible platform for testing out various algorithms. Furthermore, the convenience of being able to produce the robot on-site through 3D-printing can speed up the development process, allowing for rapid iteration and implementation of new ideas.

The switch from LiPo batteries to Li-Ion Makita batteries has markedly increased the safety of Robotont. Charging LiPo batteries is hazardous due to potential for self-combustion. During their handling and use, extra care also has to be taken to prevent accidental physical damage, which could also result in self-combustion. Moreover, even though Robotont 2 had a dedicated battery slot inside the robot body, the LiPo batteries are prone to swelling upon continued use, which rendered them too large to fit inside this compartment. Wire connection was also inconvenient inside the compartment. As a result, the batteries were most often placed on top of the robot when driving, instead of inside the battery compartment, which introduced significant potential for mechanical damage to the batteries. The use of Makita batteries for Robotont 3 eliminates these concerns, as the batteries have integrated control circuits to prevent user error while charging, a rigid chassis to prevent mechanical damage, a simple slide mechanism that requires no wire connections when replacing the battery, and they are not prone to swelling. Overall, the incorporation of Makita batteries has made Robotont safer, which is an important advancement especially in educational settings.

### 4.5 Limitations and future work

The introduction of 3D-printing has made quick prototyping and cost-effective rapid development easy. The first design of Robotont 3 relies heavily on the previous generation and aims to preserve the visual identity. In terms of fully utilising the benefits of 3D-printing in manufacturing, the design could be much more optimised. For example, the amount of flat parts and spacers could be reduced and some of the parts replaced with more complex geometric designs that would be difficult to achieve with a CNC-mill, but simple to fabricate with a 3D-printer. In the future, it would also be viable to explore other design methods, such as generative design, to explore more design avenues.

Robotont 3 chassis was designed in SolidWorks, which is a proprietary CAD software. While widely used in the engineering community, SolidWorks entails licensing fees. Both of these considerations point to areas for improvement to fully align with the principles of an open-source hardware project. SolidWorks was used because of its wide array of tools and options, as well as proven stability, both of which helped to streamline Robotont’s design. In the future, it could be beneficial to migrate the chassis development to a free, open-source CAD software such as FreeCAD or OpenSCAD.

The frame module parts are sensitive to printer differences, as the fit can easily change. This results in difficulties in assembling the chassis and can potentially necessitate some manual filing of the parts. To avoid this, a calibration set is provided (Figure 7A), which can be printed out with different settings until a suitable result is obtained. However, more features could be added to the calibration pieces to account for more possible failure modes.

The lattice structures used throughout the chassis provide a weight reduction and potential increase in durability, but at the same time the holes allow objects to fall into the structure, potentially damaging the printed circuit board or the connections. In the future, a more closed chassis design should be tested to provide more protection for the electronics.

Consolidating all electronics onto a single PCB sacrifices modularity, as enhancements to one part necessitate manufacturing the entire board. Additionally, while two power input connectors are present, the current implementation of power path switching allows only one input at a time. Connecting two supplies with differing voltage levels risks reverse currents through body diodes of high-side switches, potentially damaging MOSFETs or overloading power sources. This issue will be addressed in the next iteration of the mainboard, either by incorporating additional components or by implementing a dedicated power switching chip.

The power management subsystem is currently being developed in the Arduino framework, chosen for its convenience in verifying prototype functionality. However, transitioning to a more optimized C-based framework is anticipated as the system evolves and features become more finalised.

The firmware architecture provides a robust foundation for programming the main MCU. However, there are essential functionalities yet to be developed, such as establishing a standardised communication protocol for interfacing with additional devices and facilitating seamless data exchange with the onboard computer.

## Data Availability

The datasets presented in this study can be found in online repositories. The names of the repository/repositories and accession number(s) can be found in the article/Supplementary Material.

## Supplementary material

SUPPLEMENTARY DATA SHEET S1 Bill of materials.

SUPPLEMENTARY DATA SHEET S2 Assembly instructions.
