# Supplementary material for: Robotont 3–an accessible 3D-printable ROS-supported open-source mobile robot for education and research
Source: Front Robot AI. 2024 Jul 10;11:1406645. doi: 10.3389/frobt.2024.1406645 (PMC11265998; doi:10.3389/frobt.2024.1406645)

ROBOTONT

assembly instructions

Robotont version: 3.0
Date: 2024-03-21

Table of contents

Cabling	3
PCB assembly	7
3D printing	16
Battery module assembly	18
Robot assembly	26

Cabling

All cables needed for Robotont

x1
on-board computer
power cable

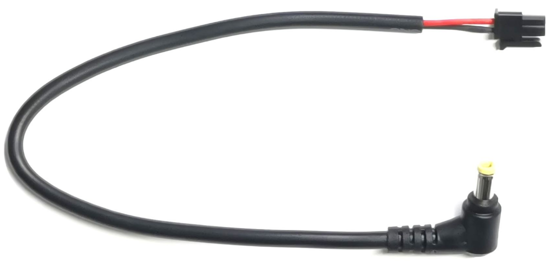

x1
STOP switch
cable

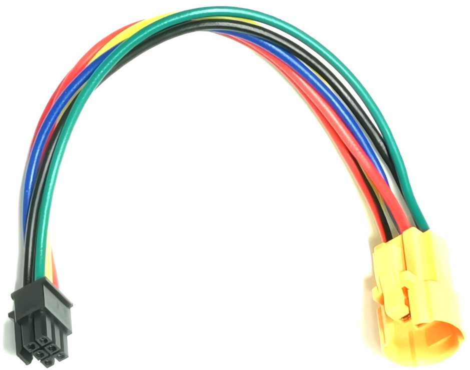

x2
USB Type C
data cables

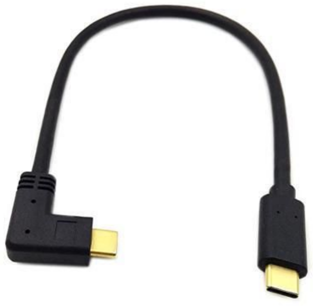

Onboard computer power cable

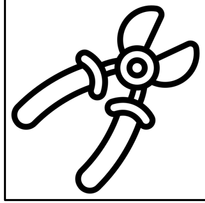

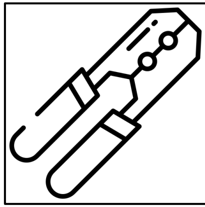

- Cut the cable to 150 mm from the right angled barrel plug
- Crimp Molex Micro-Fit terminals to the wires
- Insert terminated wires to 2x1 pos Micro-Fit 3.0 female connector
    - Black wire (GND) goes to the side next to the locking fastener

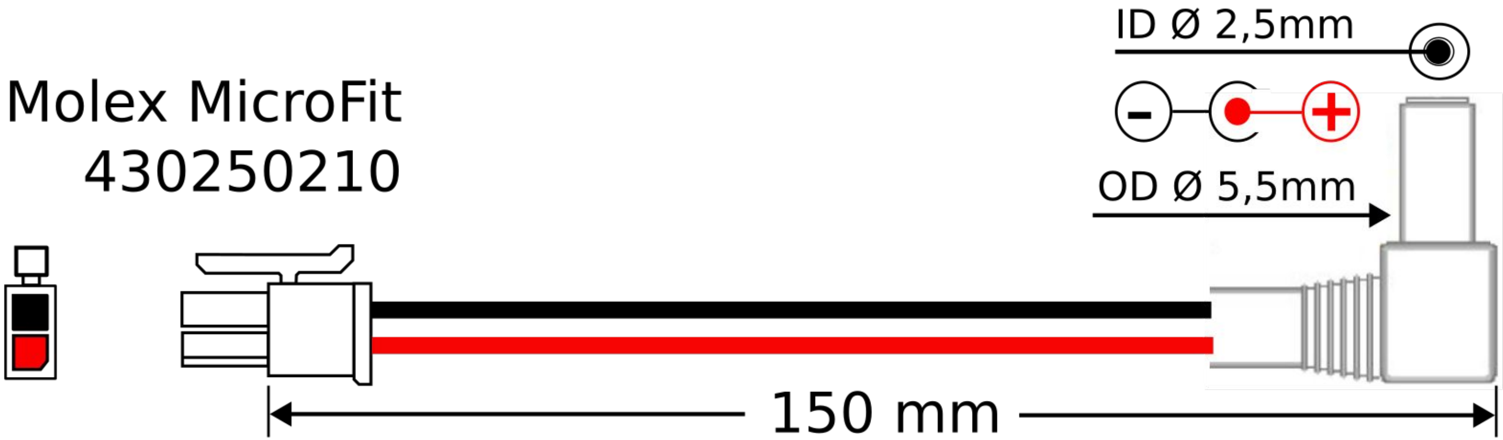

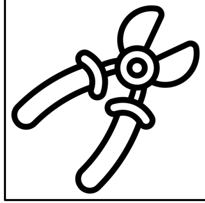
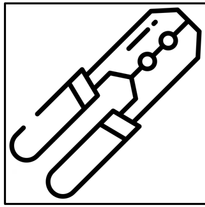

Stop switch cable

- Cut the stop switch wires to 200 mm length
- Crimp Molex Micro-Fit terminals to all wires
- Insert to 2x3 pos Micro-Fit 3.0 female connector
    - Follow the numbering below for the correct mapping

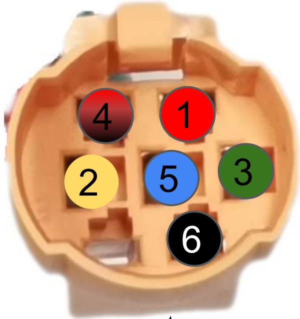

1 - LED Common (3.3 V)
2 - Switch Normally Closed
3 - Switch Common (3.3 V)
4 - LED Red -
5 - Switch Normally Open (not used)
6 - LED Green -

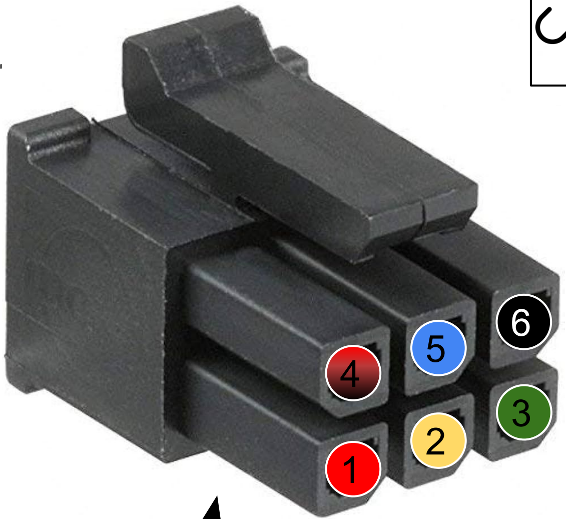

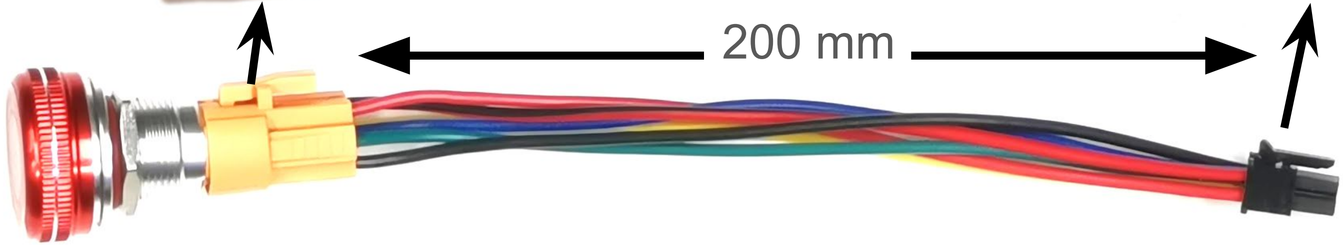

PCB assembly

Cut the mouse bites

Bend the sides

Remove the outer manufacturing panel

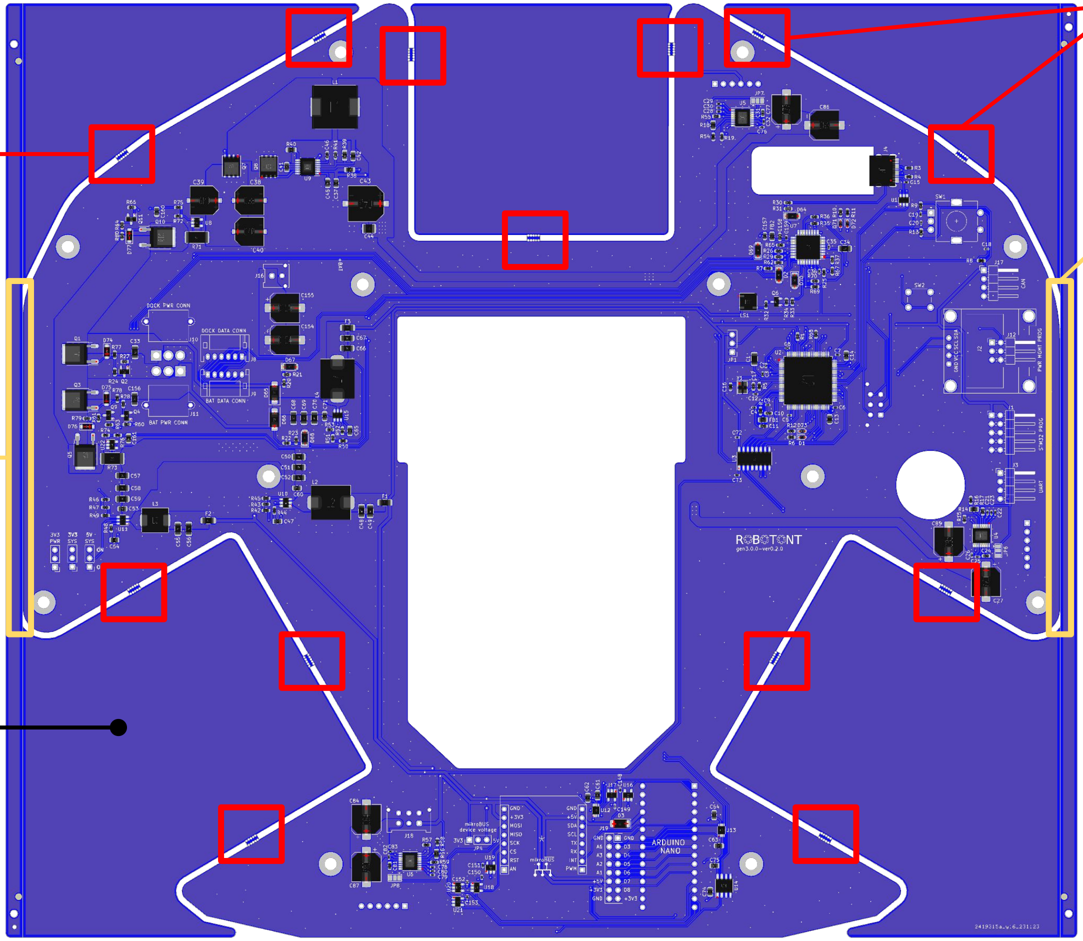

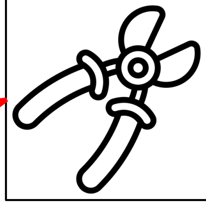

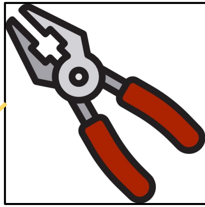

Solder 1x3 pinheader for MikroBUS voltage selection

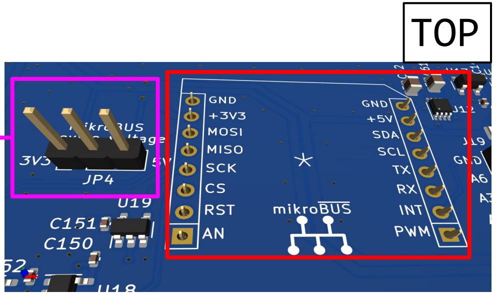

Solder MikroBUS dedicated socket (alternatively two 1x8 pinsockets)

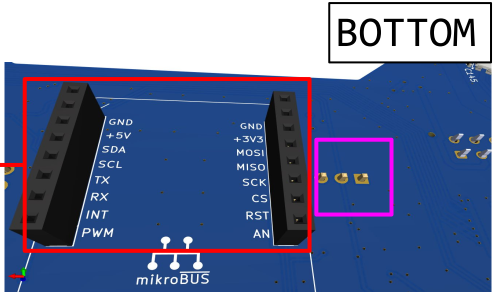

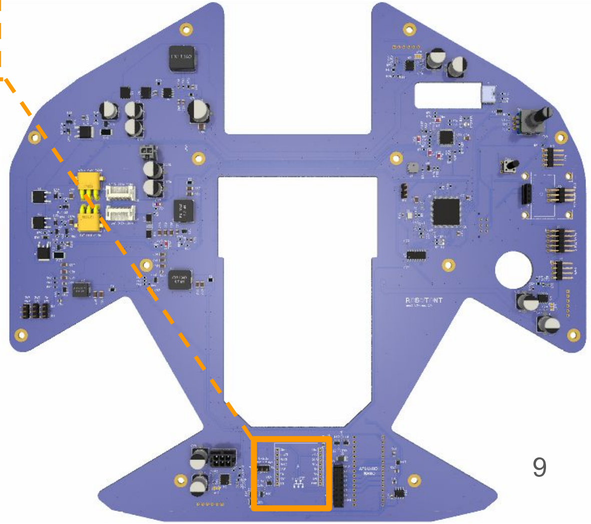

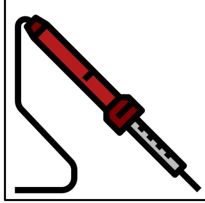

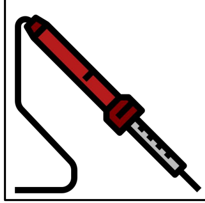

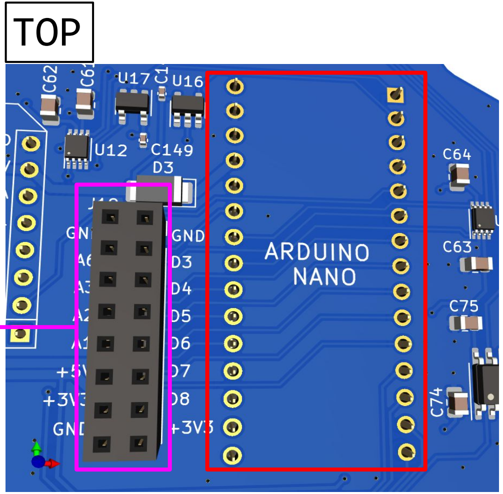

Solder 2x3 pinsocket for accessing unused pins

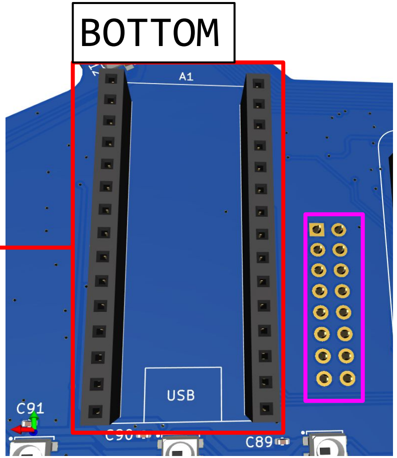

Solder two 1x15 pinsockets for Arduino

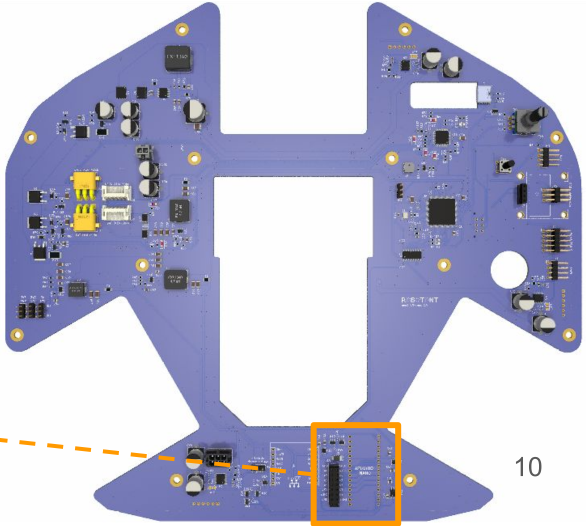

Solder 2x3 Micro-Fit socket
for the stop switch

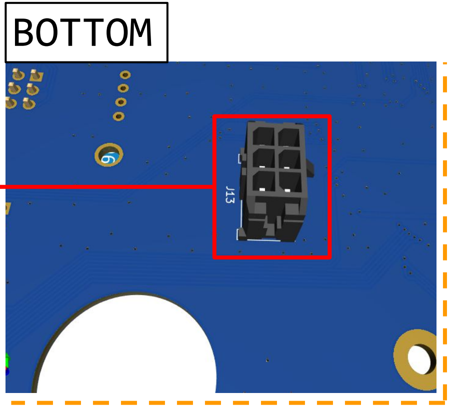

Solder 2x1 Micro-Fit socket for
onboard computer power supply

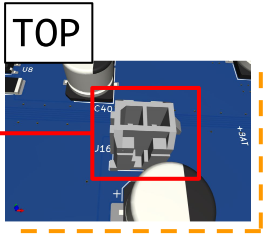

Solder 2x3 Micro-Fit socket
for external power

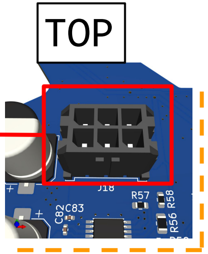

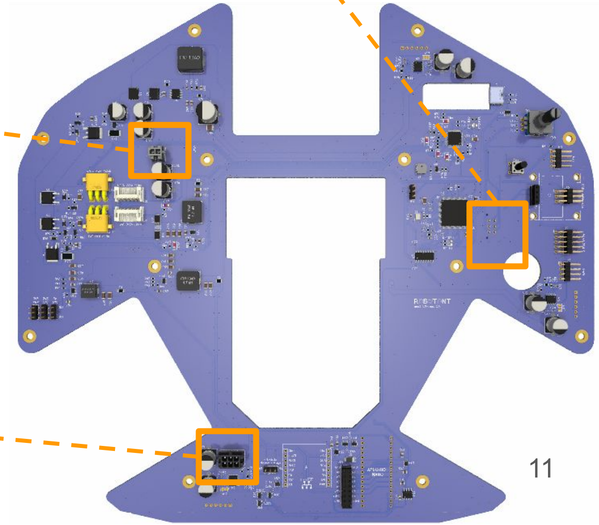

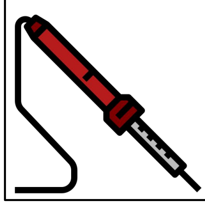

Solder 2x3 right-angled pinheader for the power management MCU programming

Solder 2x5 right-angled pinheader for the main MCU programming

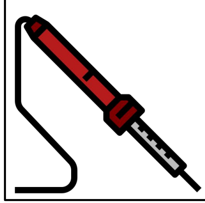

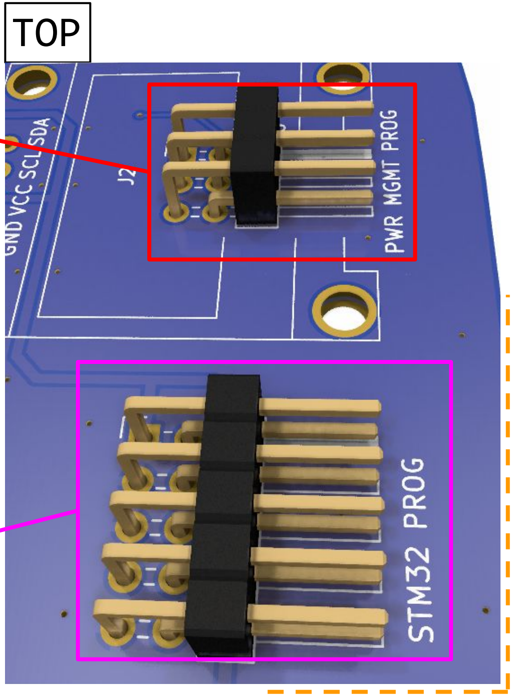

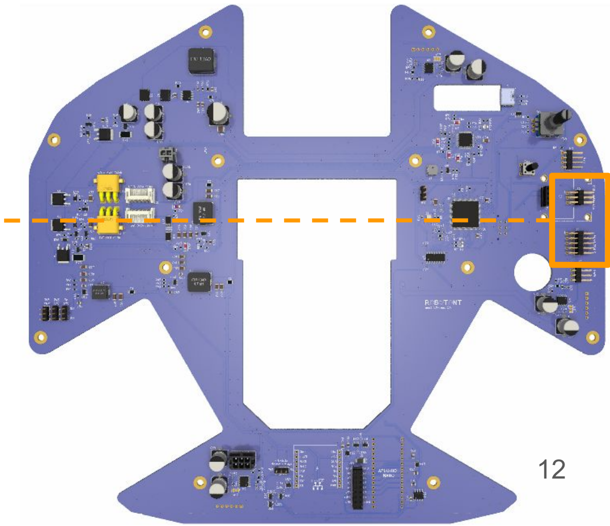

TOP

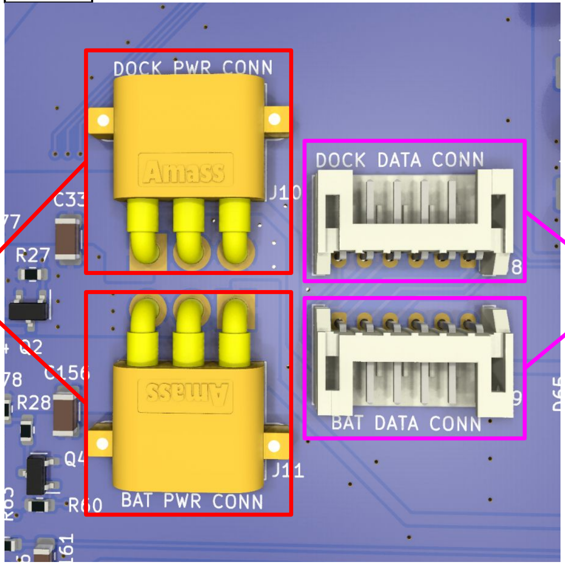

Solder two MR30PW male power connectors

Solder two JST-PH-6 male data connectors

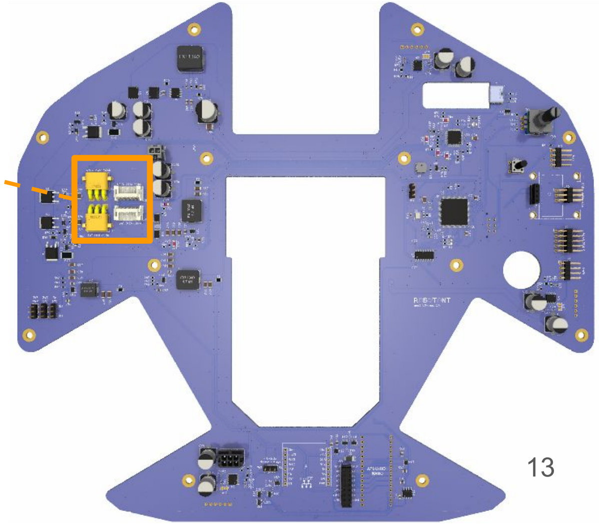

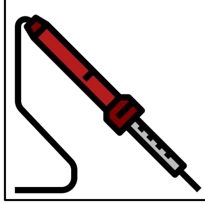

Solder 1x4 pinsocket for the OLED display

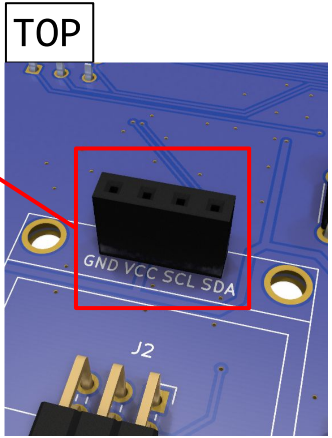

Solder three 1x6 angled pinheaders for the motors

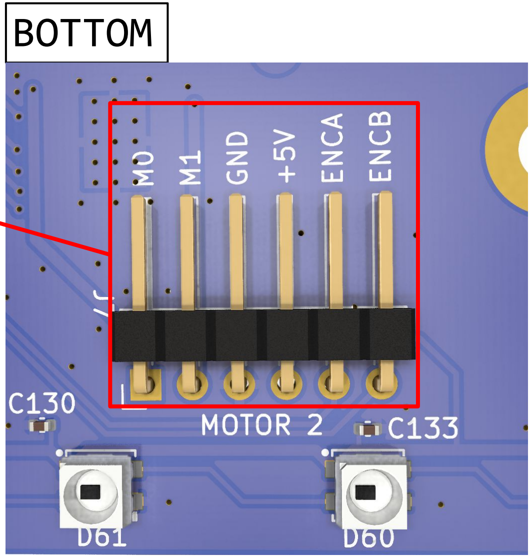

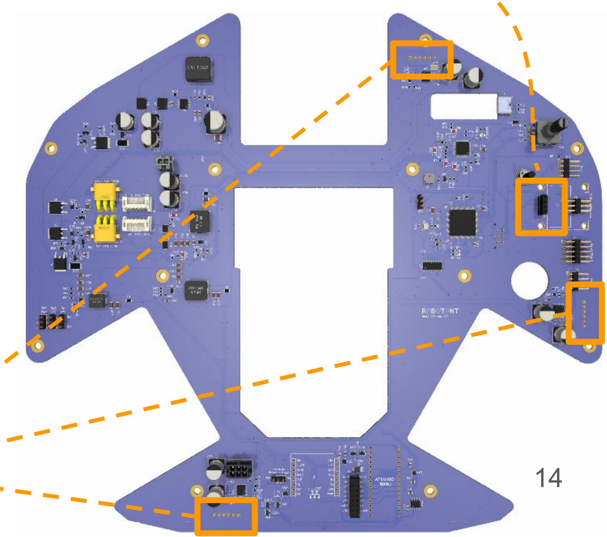

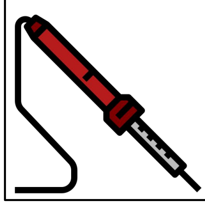

Solder the rotary encoder

Solder the power switch

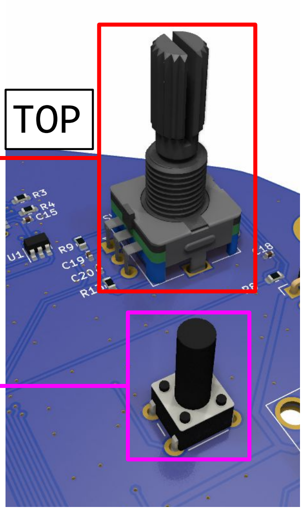

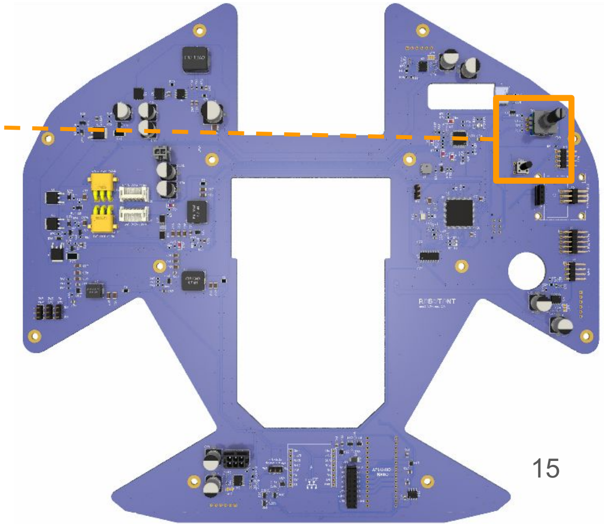

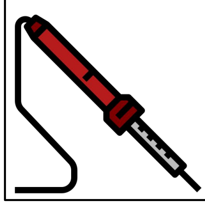

3D printing

3D printing

Print every detail in the amount shown.

Material suggestion:

- PETG for motor modules
- PLA for everything else

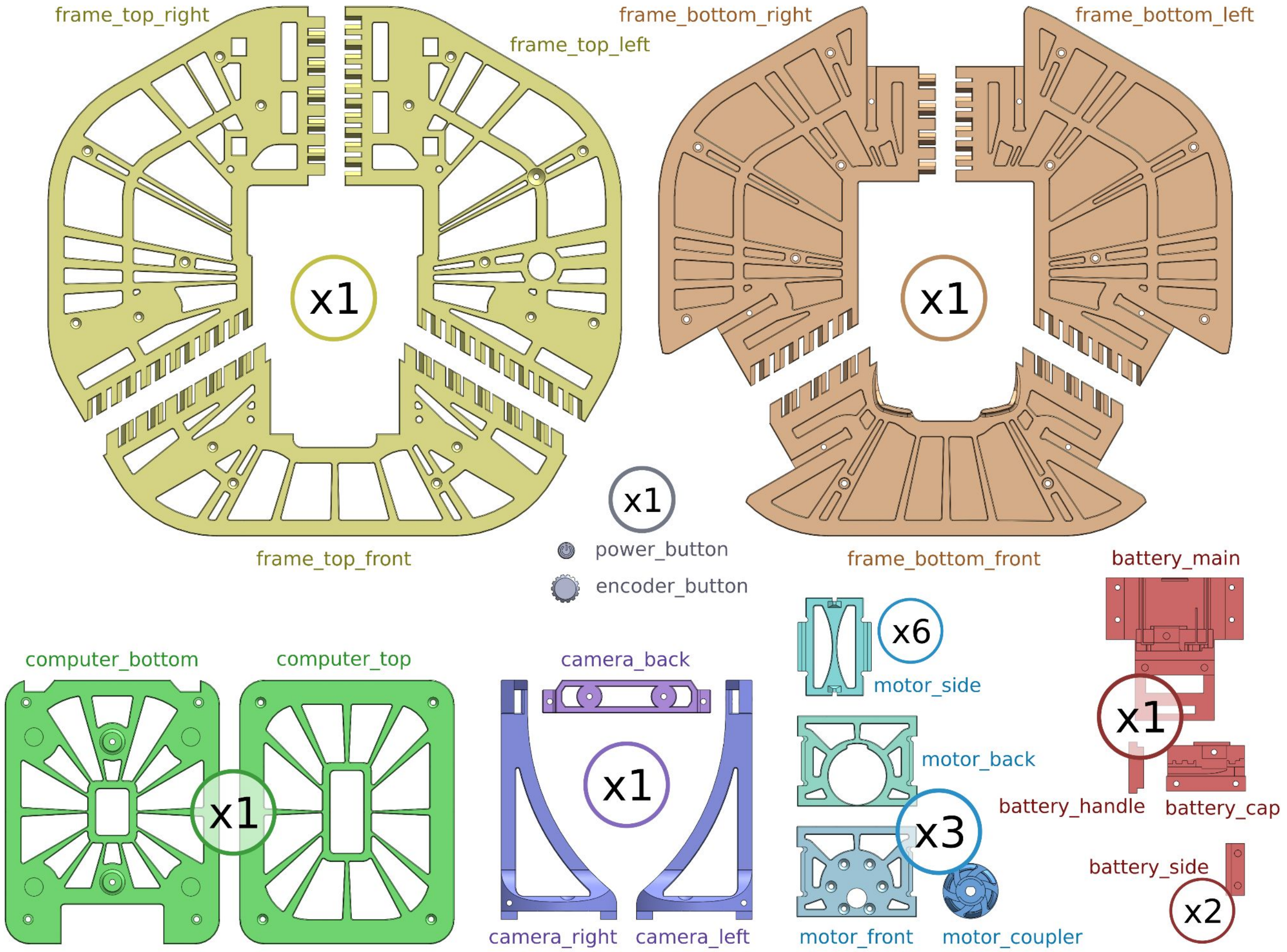

Battery module assembly

Battery module part list

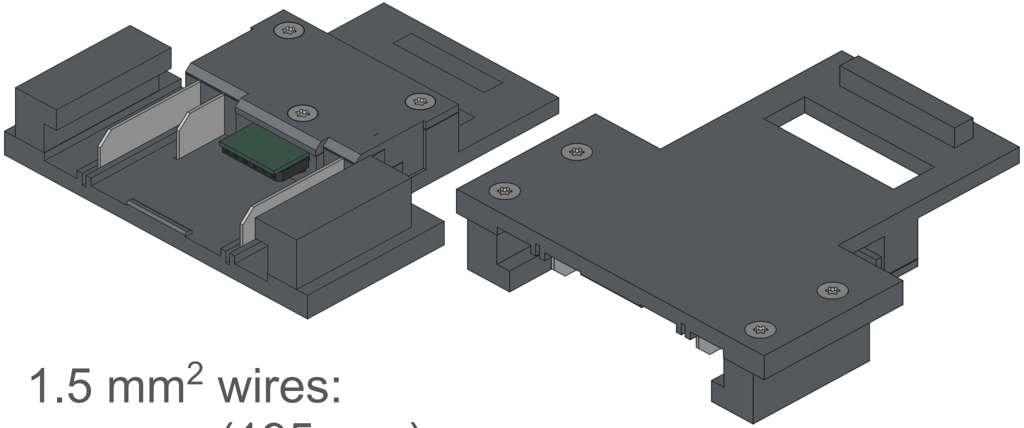

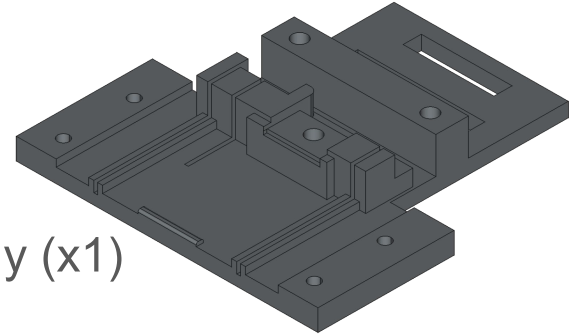

main body (x1)

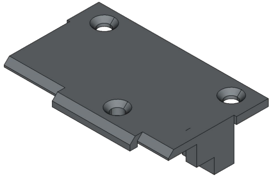

cap (x1)

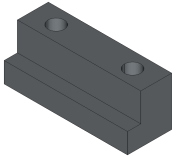

side (x2)

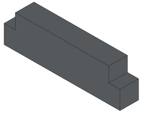

handle (x1)

1.5 mm^2 wires:

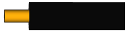 (195 mm)

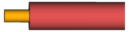 (170 mm)

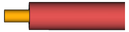 (160 mm)

0.13 mm^2 wires (x6):

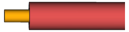 (180 mm)

AMASS MR30-FB connector (x1)

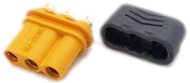

M3x8 bolts (x7)

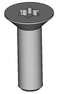

M3x5.9 heat-set inserts (x7)

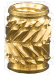

JST-PH-6
- terminals (x6)

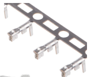

- connector (x1)

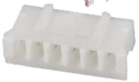

PCB + spring contacts (x1)

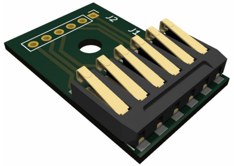

Use a soldering iron to attach M3x5.9 heat-set inserts (x7) into:

- 3 holes in the main body
- 2 holes in each side holder

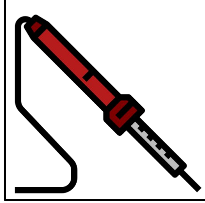

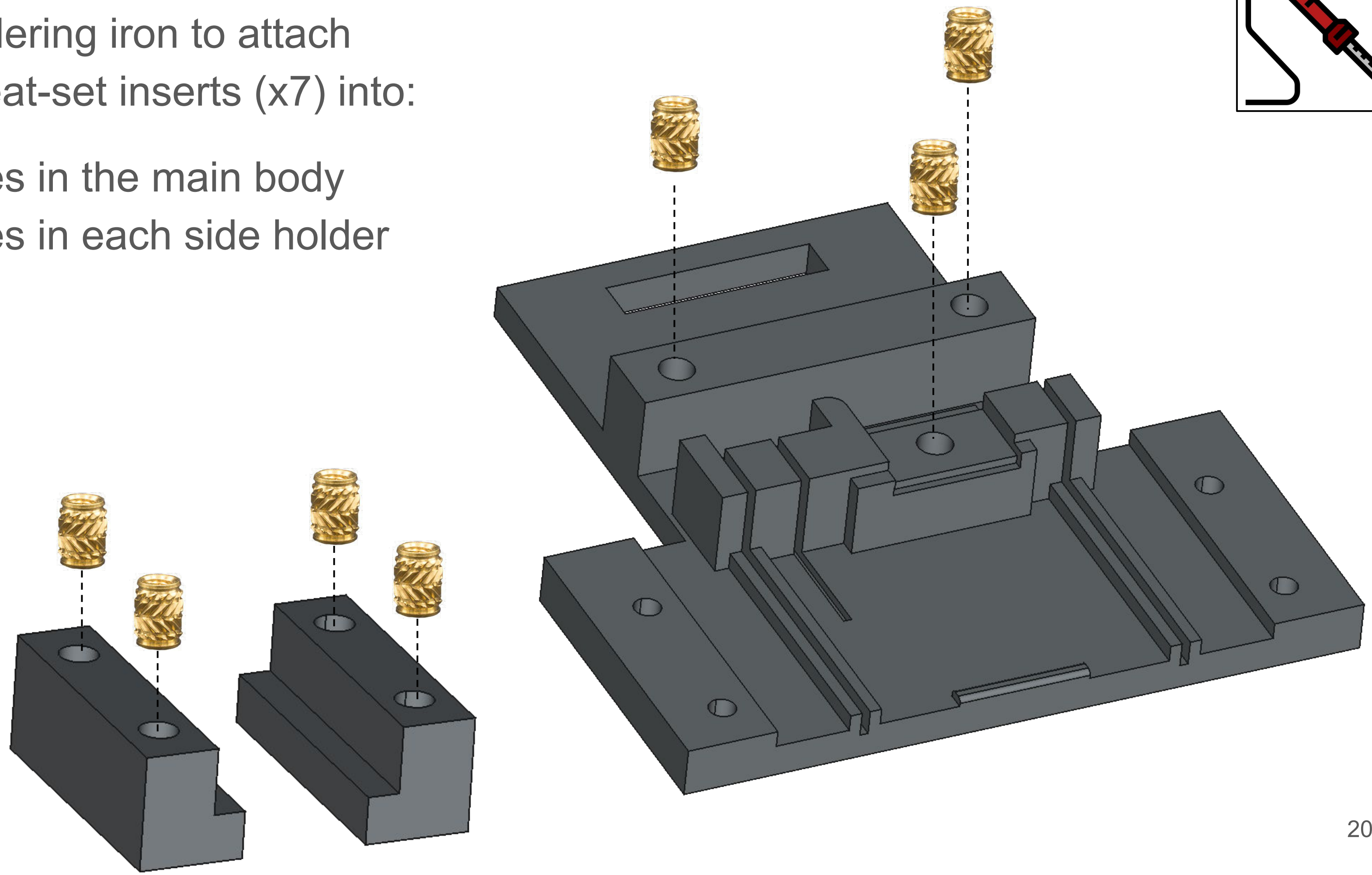

Cut contact strips from
0.7 mm thick sheet metal:

- GND: 11x40 mm
- CHG: 11x25 mm
- VCC: 11x40 mm

Bend one end of each strip by 90 degrees at 4 mm.

File or cut the corners for smoother insertion to the battery.

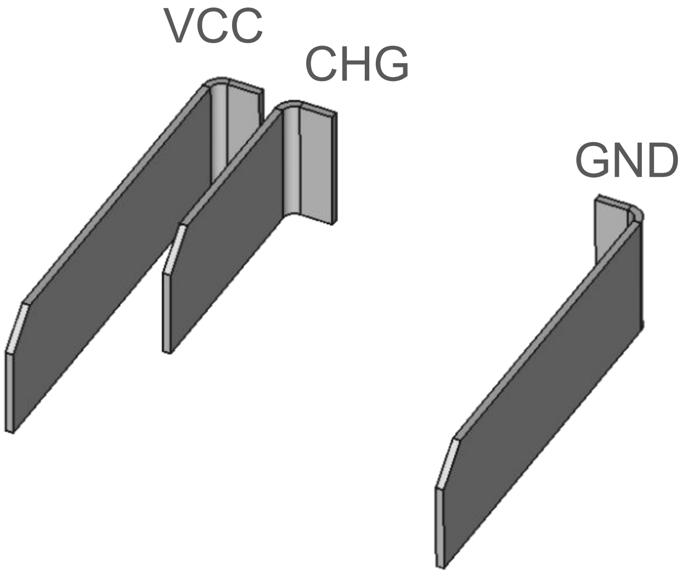

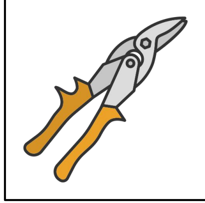

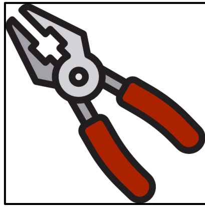

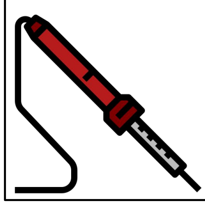

Insert the bent metal sheet contacts to the 3D-printed body

Cut and strip three cables (1.5 mm^2):

- GND (black): 195 mm
- CHG (red): 170 mm
- VCC (red): 160 mm

Solder the cables to the top of the bent area

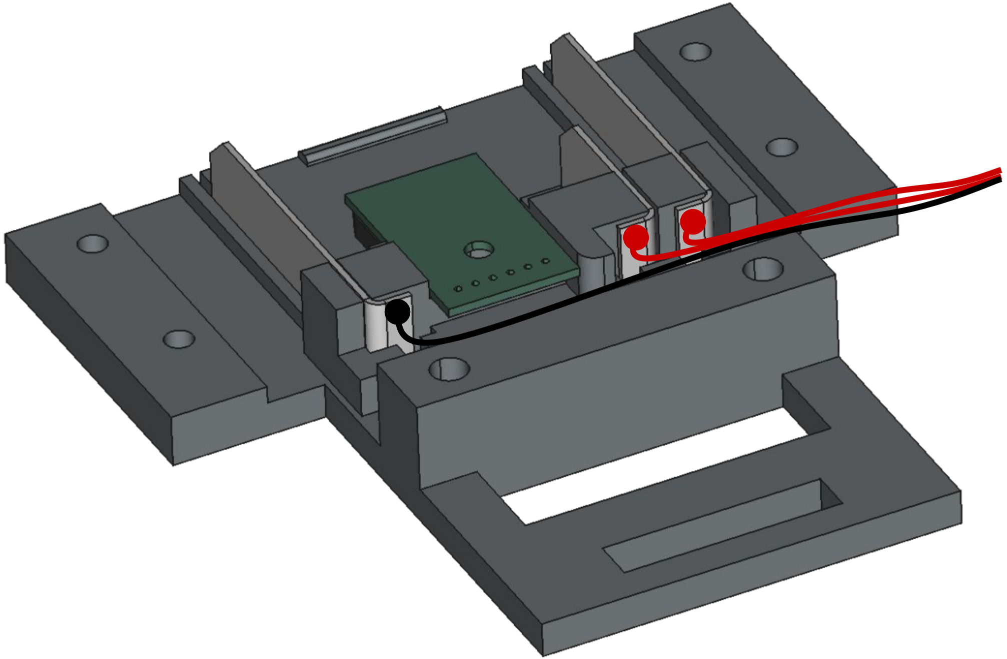

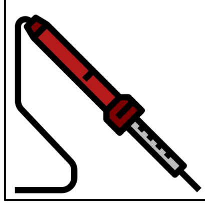

- Attach a rubber cover to the three wires
- Solder the wires to the MR30-FB female connector.
    - You will most likely need to trim the wires a little to the correct length.
- Snap the rubber cover over the connector using pliers

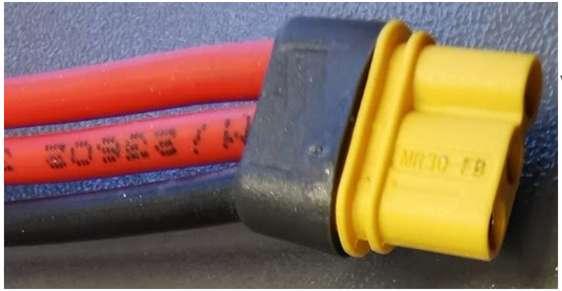

VCC
CHG
GND

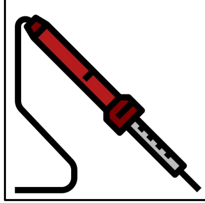

- Cut and strip six AWG 26 (0.13 mm2) cables
    - Length 180 mm
- Solder the spring contacts to the PCB
- Solder the cables to the PCB
- Crimp a JST-PH-6 terminal to the other end of the cables
    - It is a straight mapping from the PCB to the plug
- Position the PCB to its slot with spring contacts facing down
- Guide all wires through the channel to the side

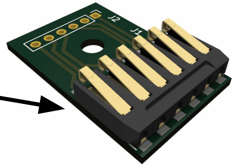

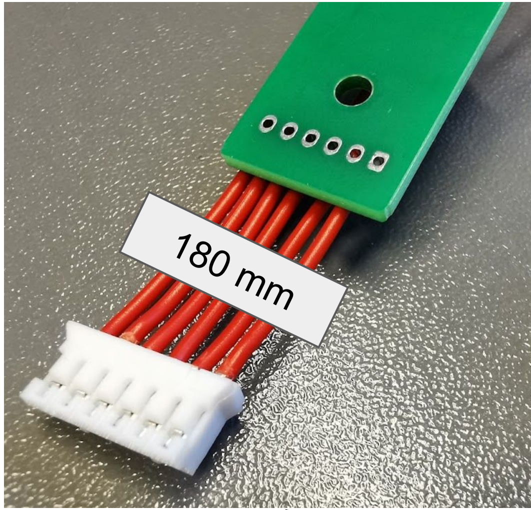

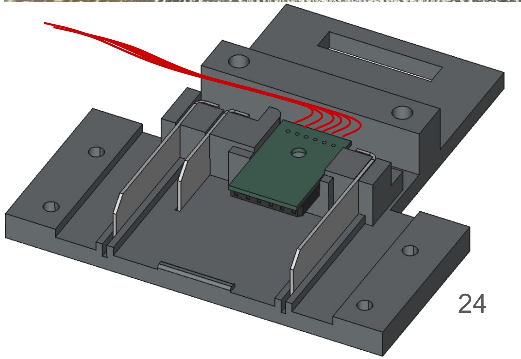

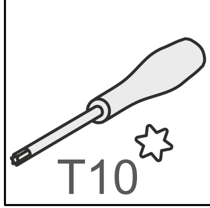

Attach the side holders and cap with M3x8 bolts (x7)

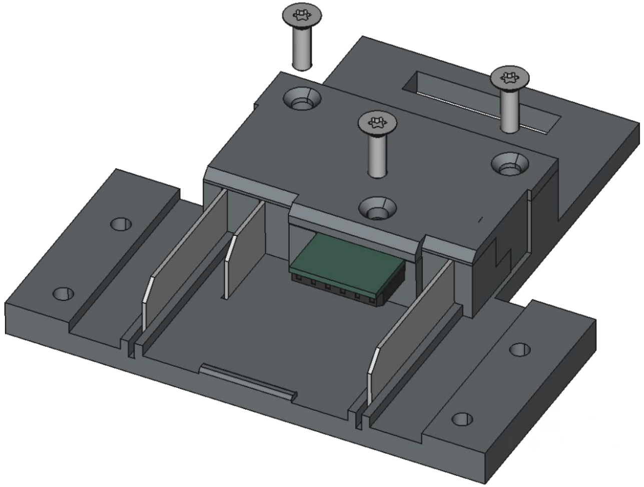

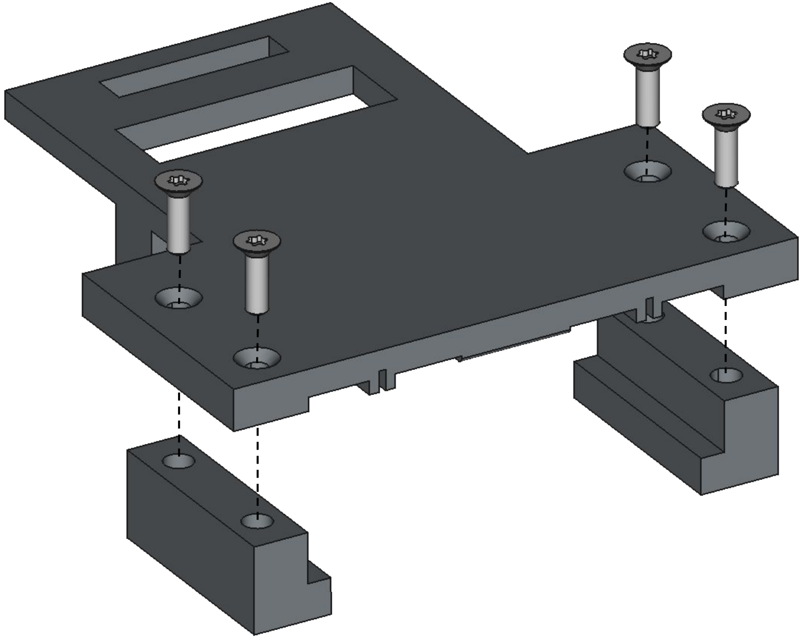

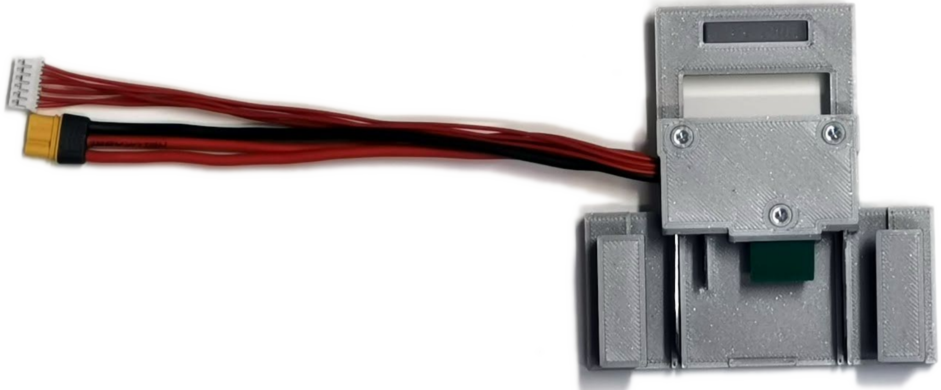

Robot assembly

Fastenings needed

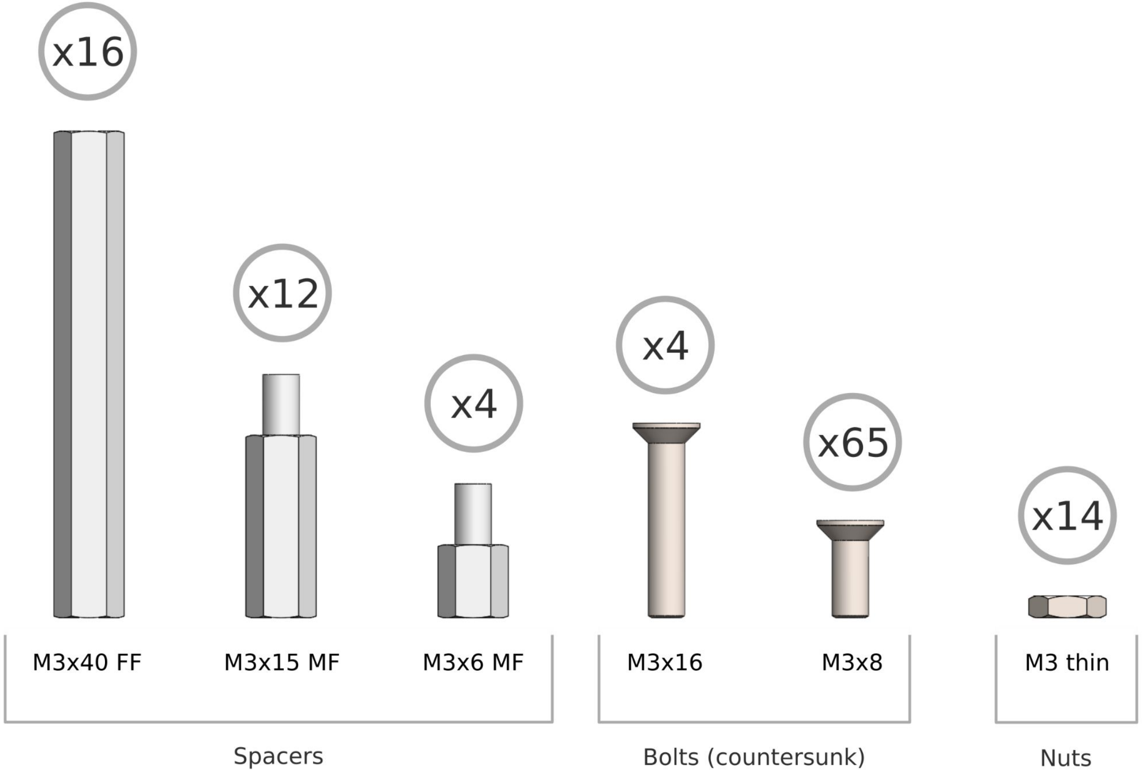

Bottom plate assembly

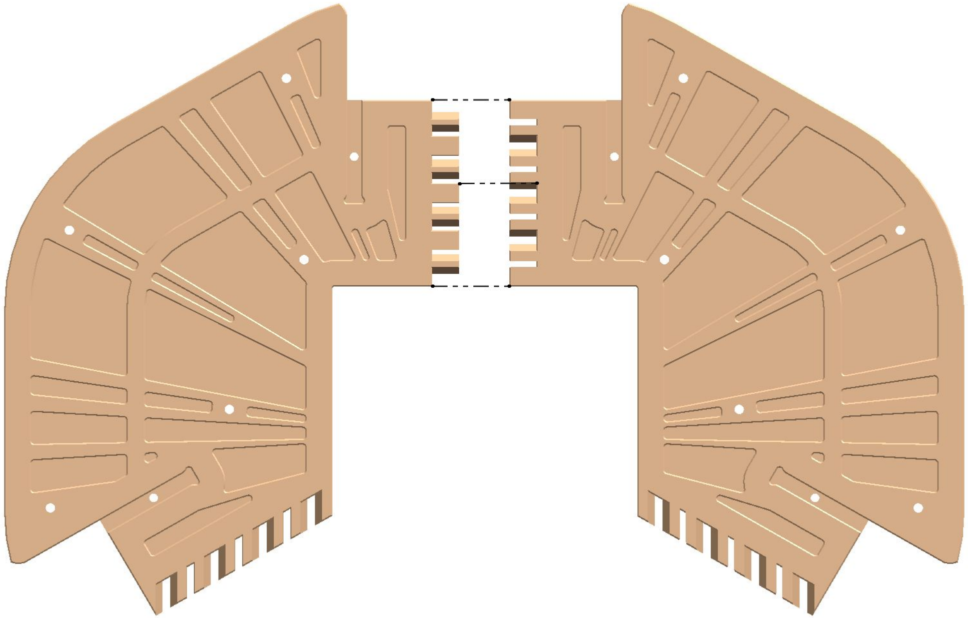

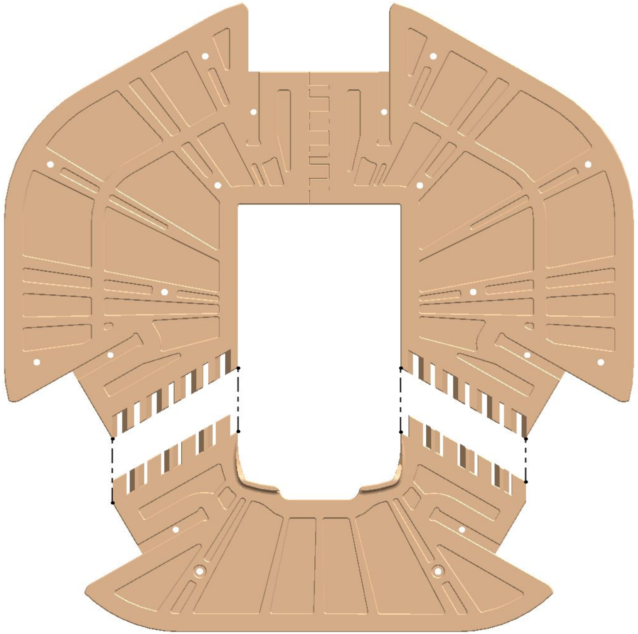

Connect M3x40 FF spacers (x12)
to bottom plate with
M3x8 bolts (x12)

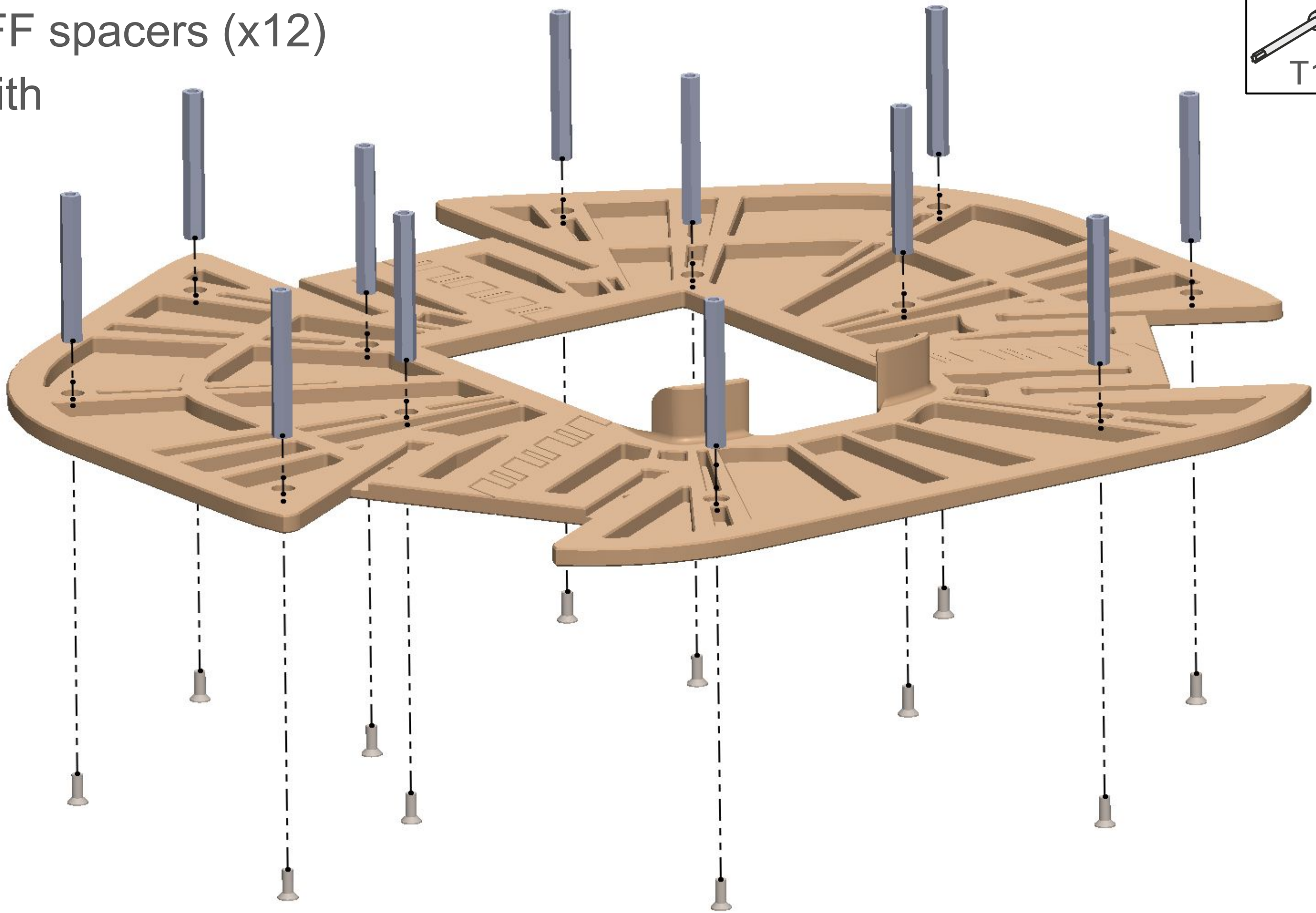

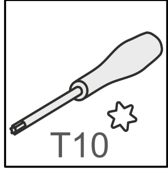

Secure PCB with
M3x15 MF spacers (x12)

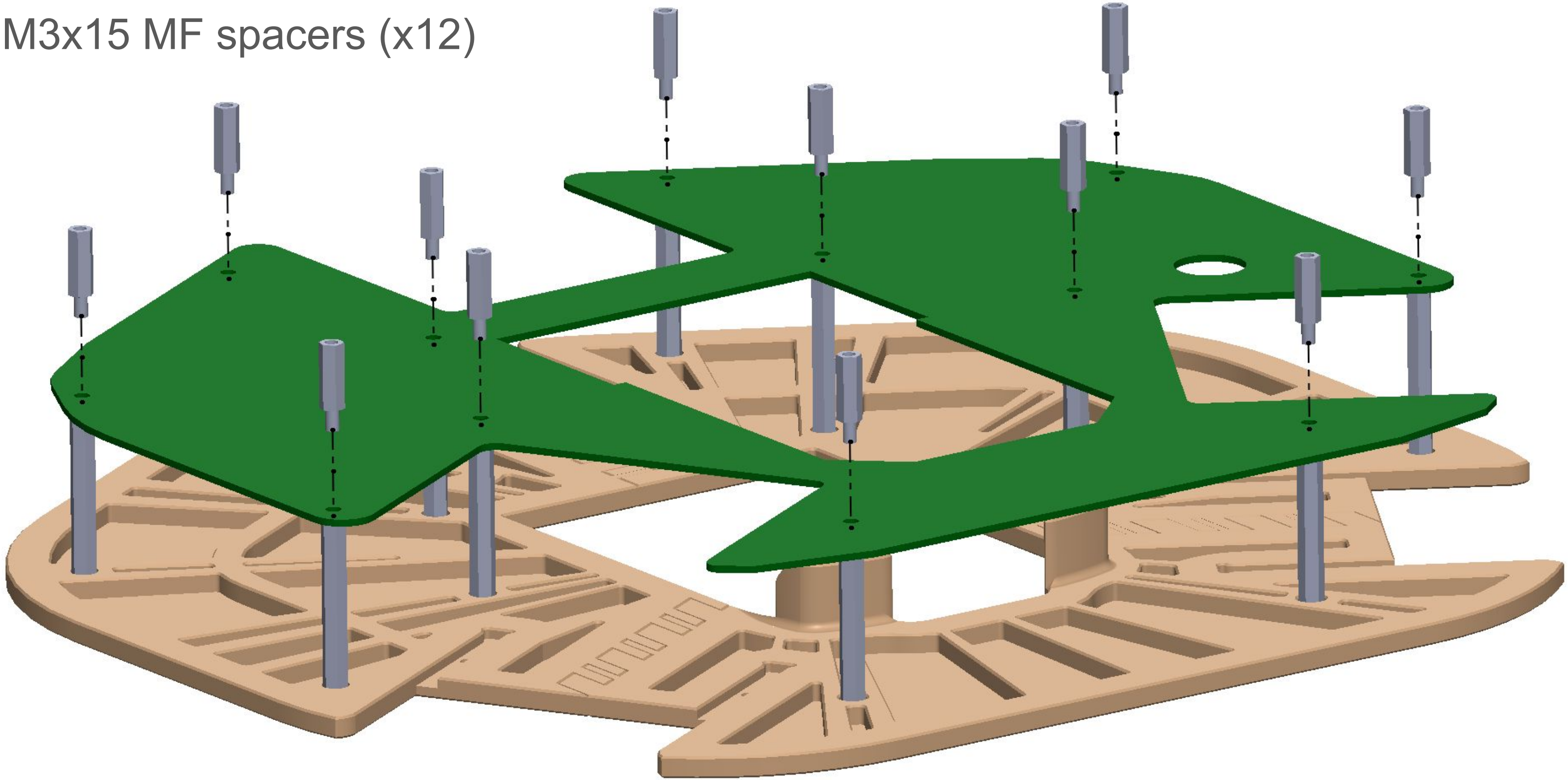

Top plate assembly

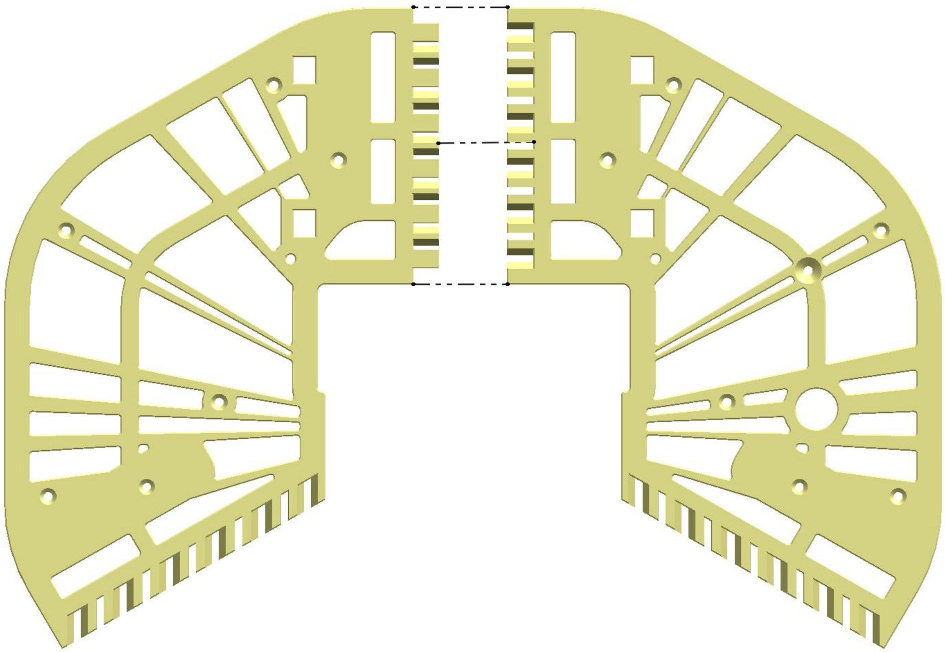

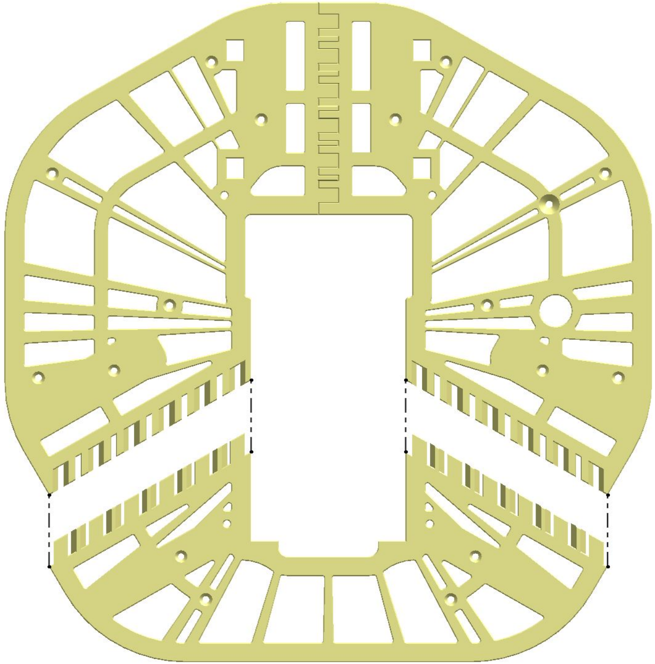

Connect E-Stop button to top plate

Secure with the flat nut

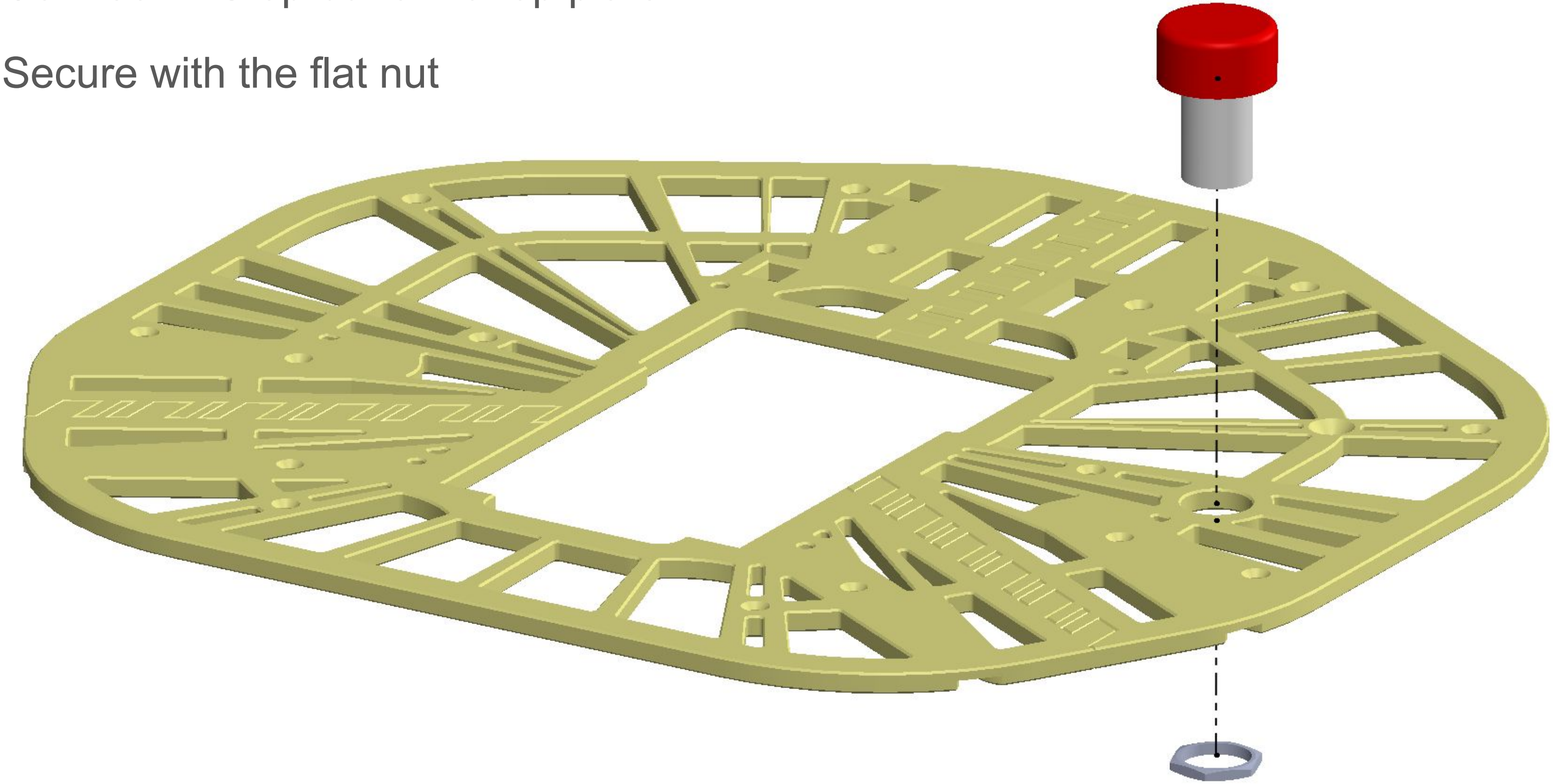

Computer module assembly

Join M3x6 MF spacers with M3x40 FF spacers in computer module bottom plate corners (x4)

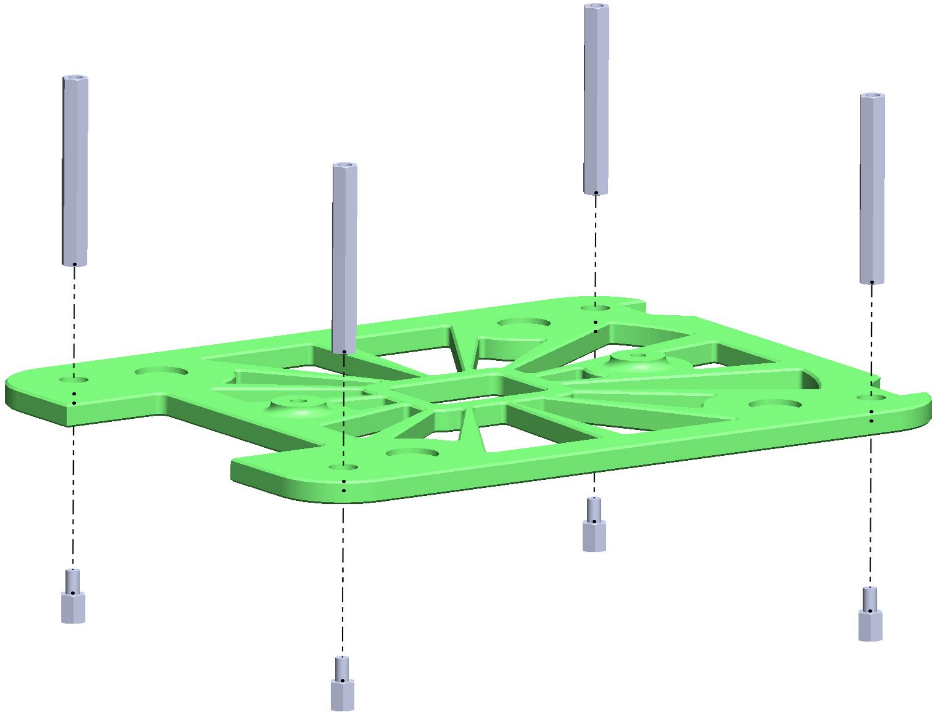

Align Intel NUC's feet with their slots (x4)

Secure Intel NUC with M3x8 bolts (x2)

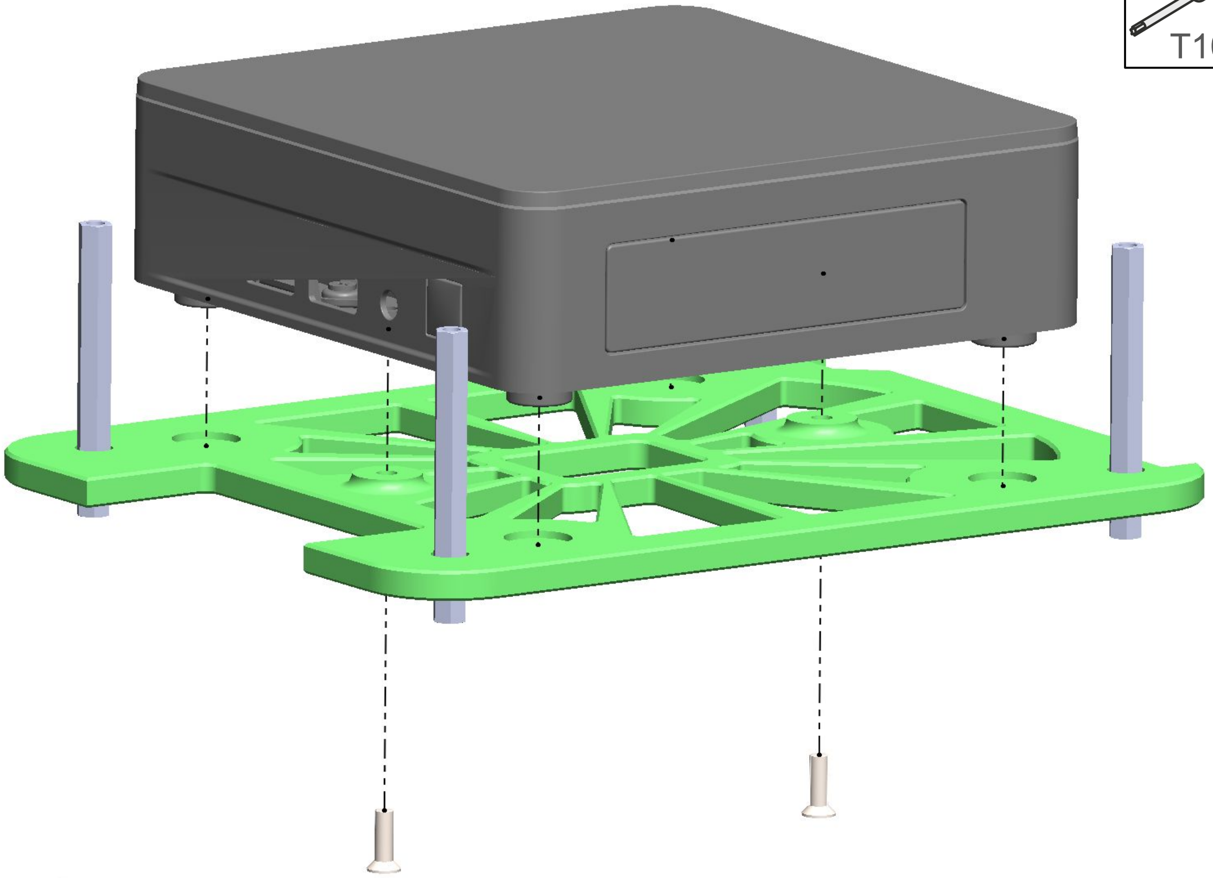

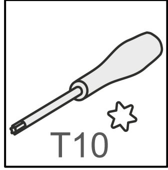

Connect computer module top plate with M3x8 bolts (x4)

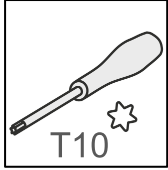

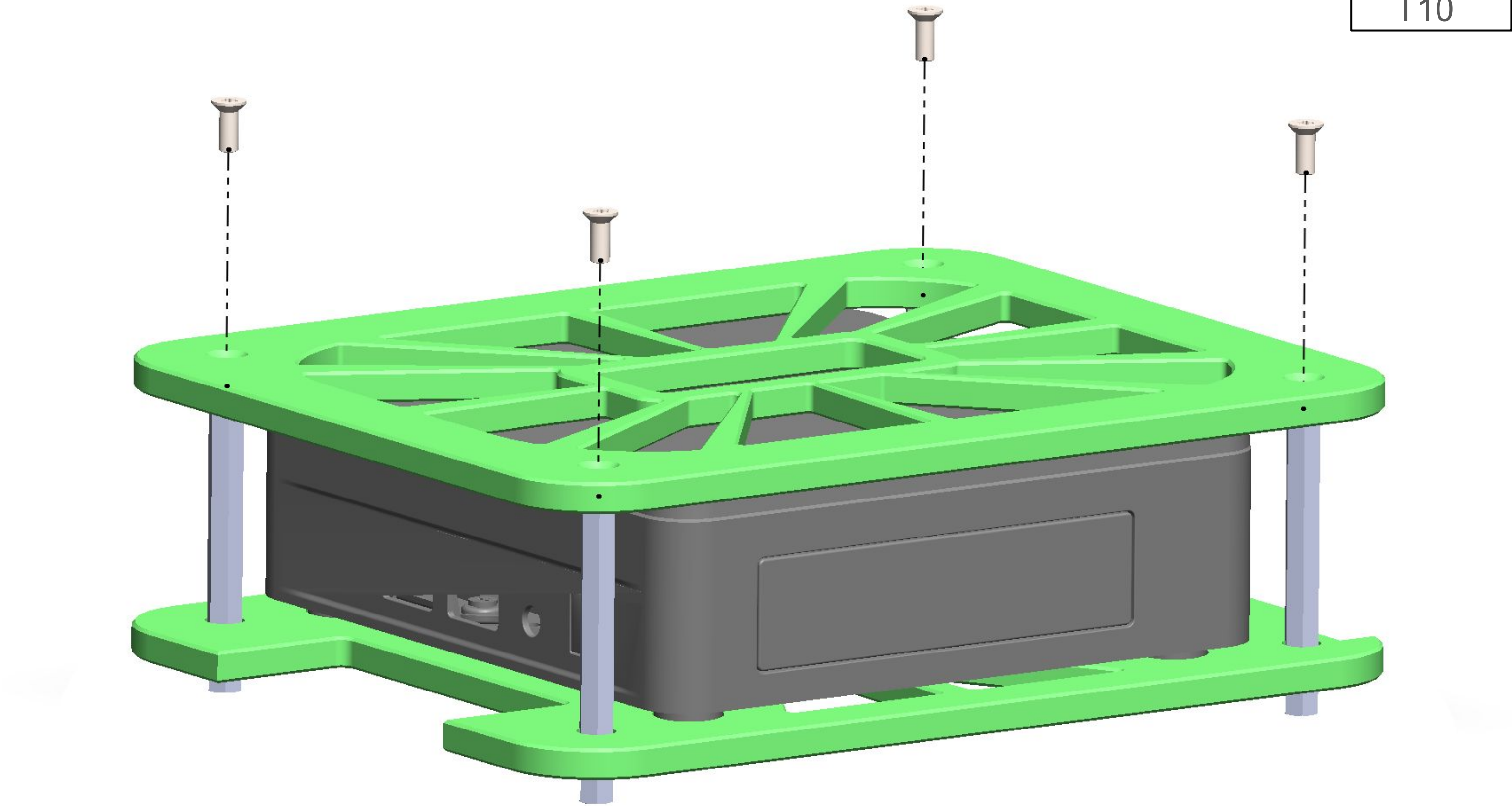

Connect the battery module to the mainboard

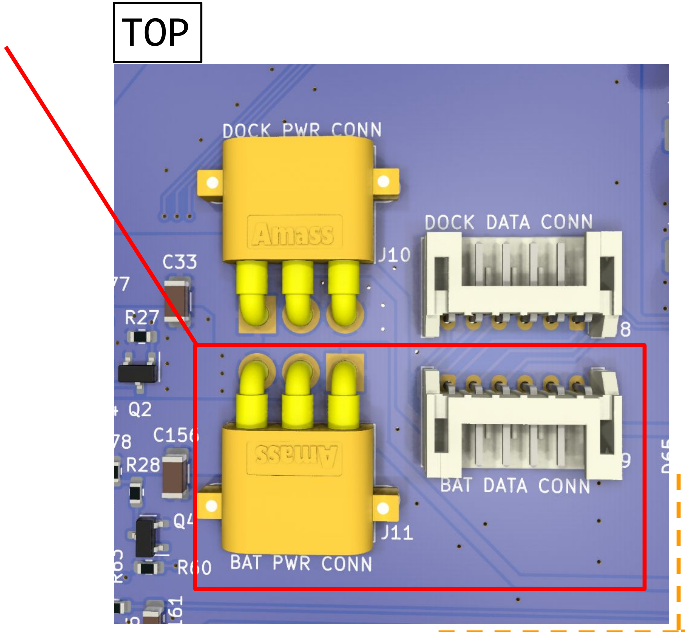

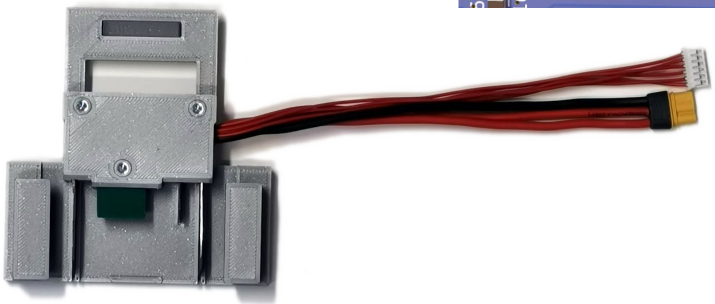

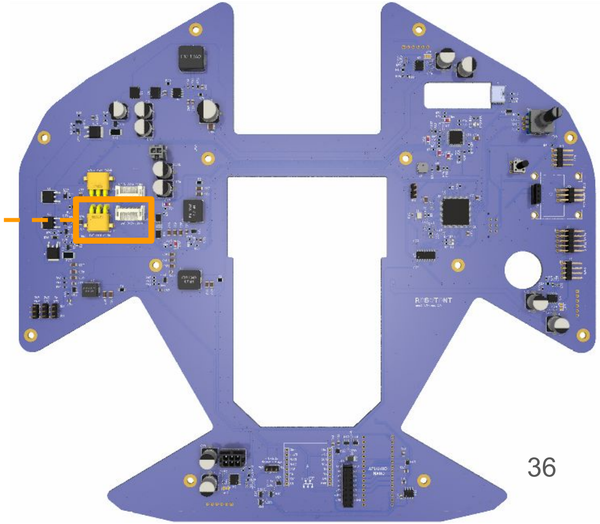

Place battery module on frame module top plate

Secure it by joining computer module to frame module top plate with M3x8 bolts (x4)

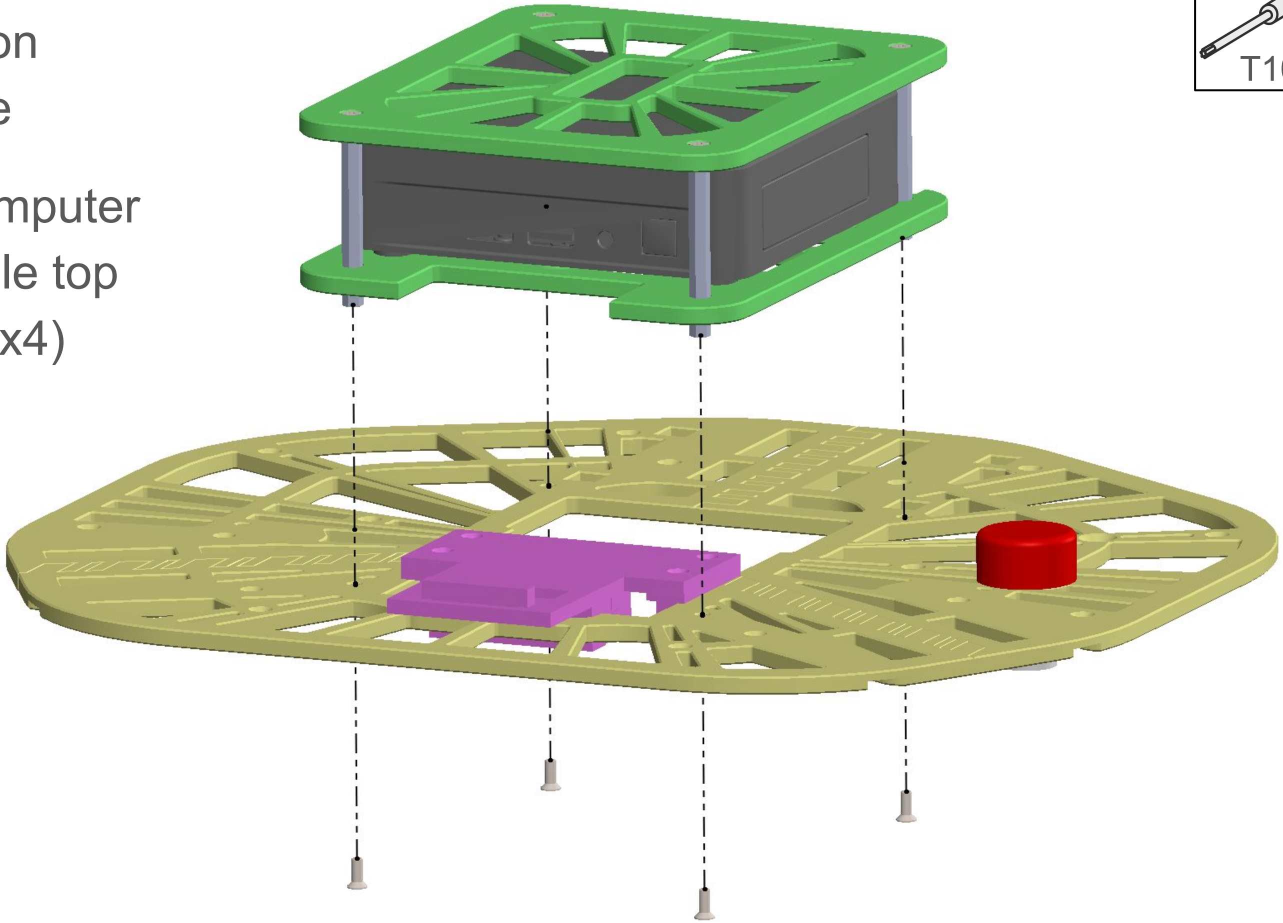

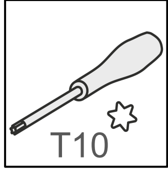

Connect bottom and top parts of frame module with M3x8 bolts (x10)

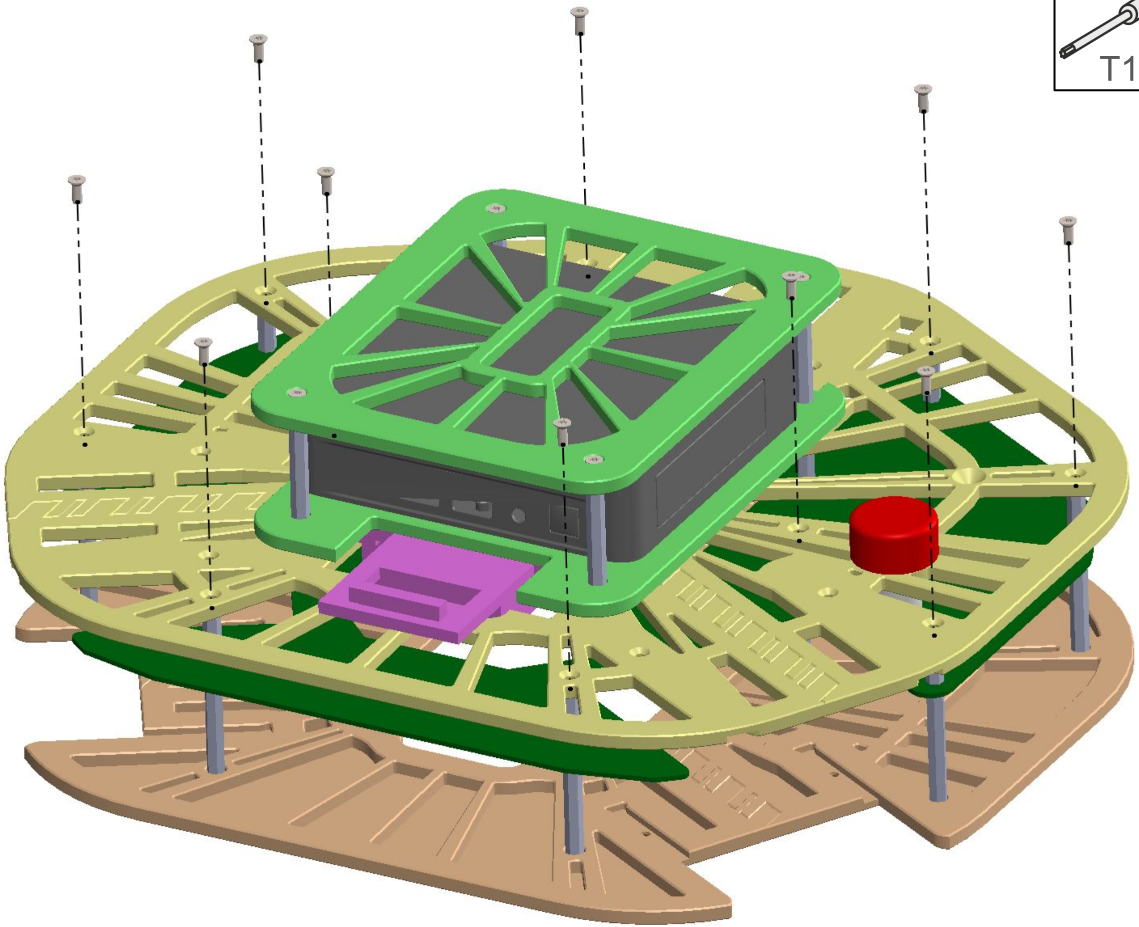

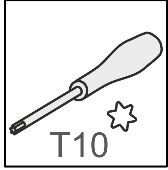

Motor module assembly (x3)

Press wheel coupler into omniwheel

It will click once attached

It only goes in from one side

NB! Align the rim cutouts with the coupler

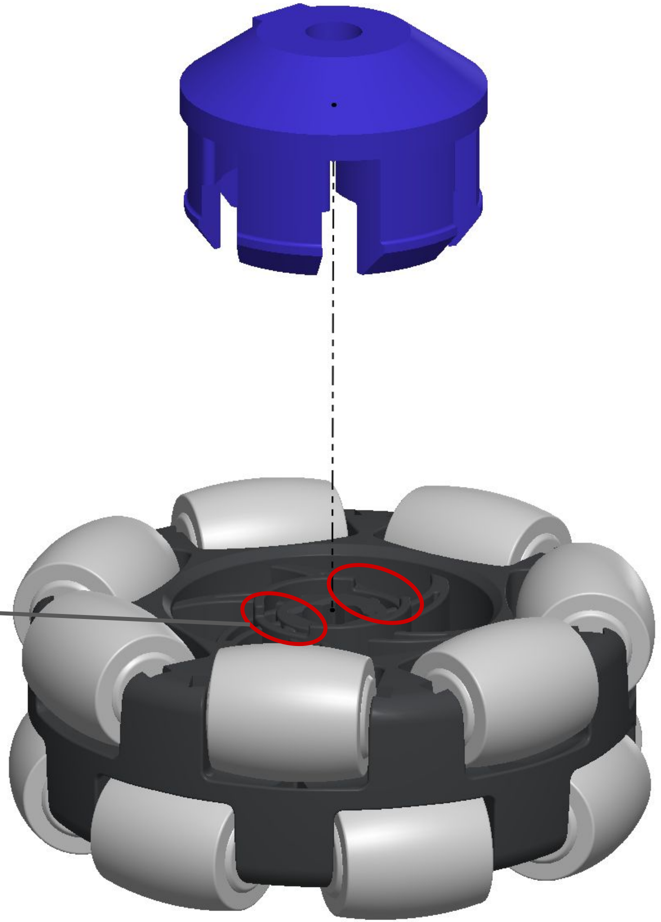

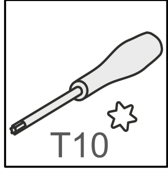

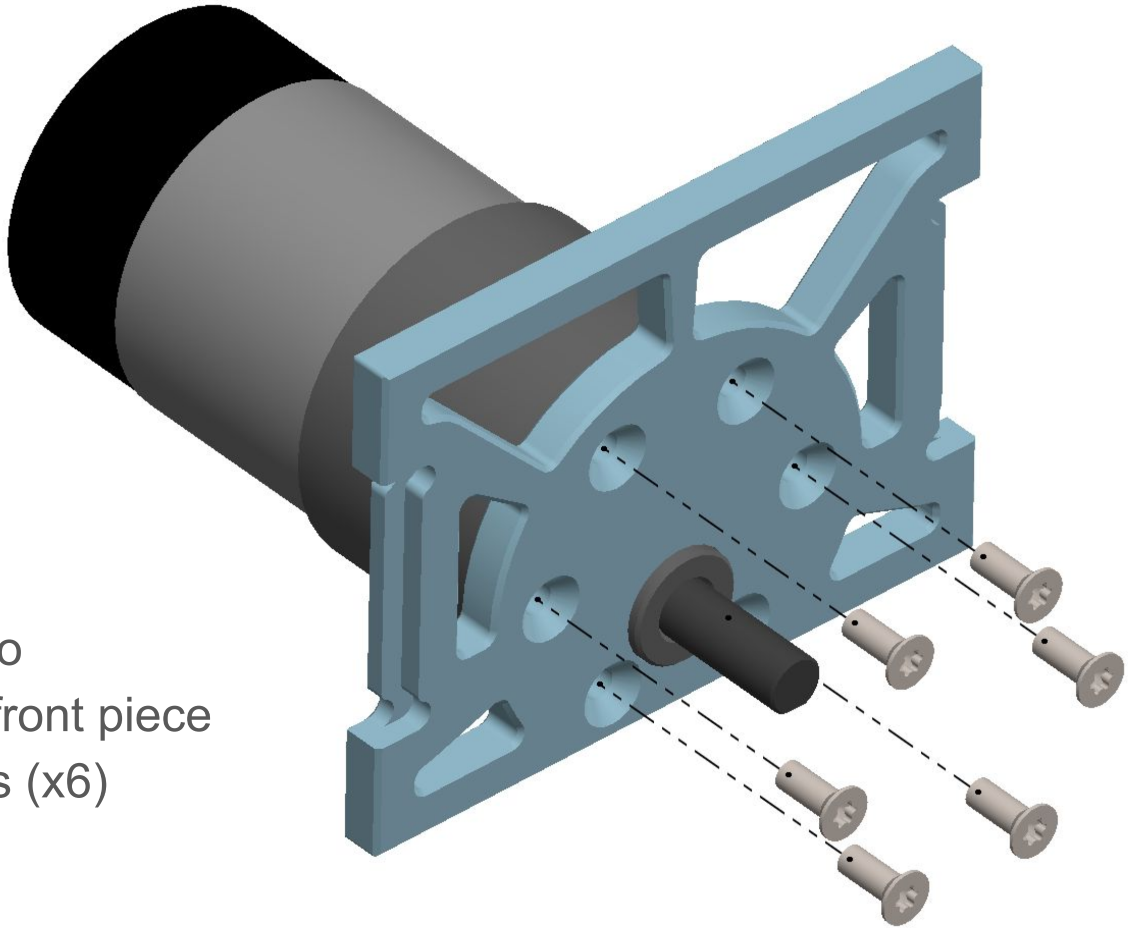

Secure motor to
motor module front piece
with M3x8 bolts (x6)

Press M3 nuts (x4) into motor module sides

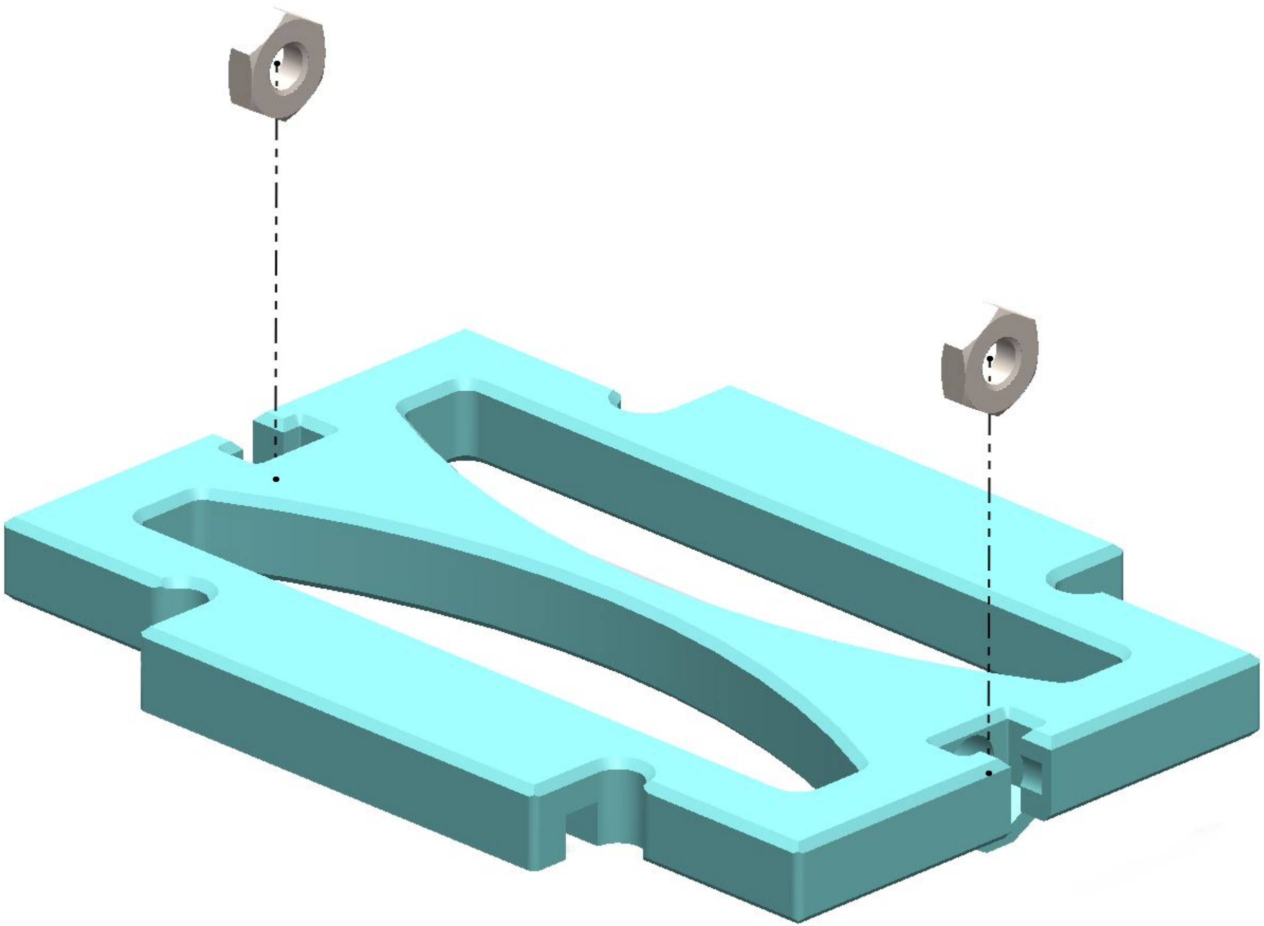

Add motor module back piece

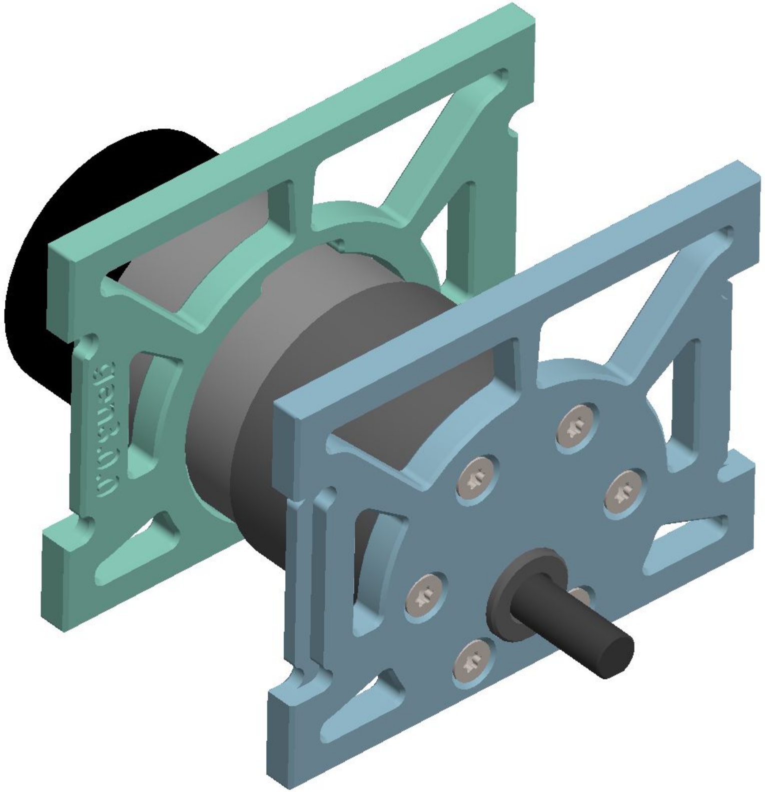

Add motor module sides

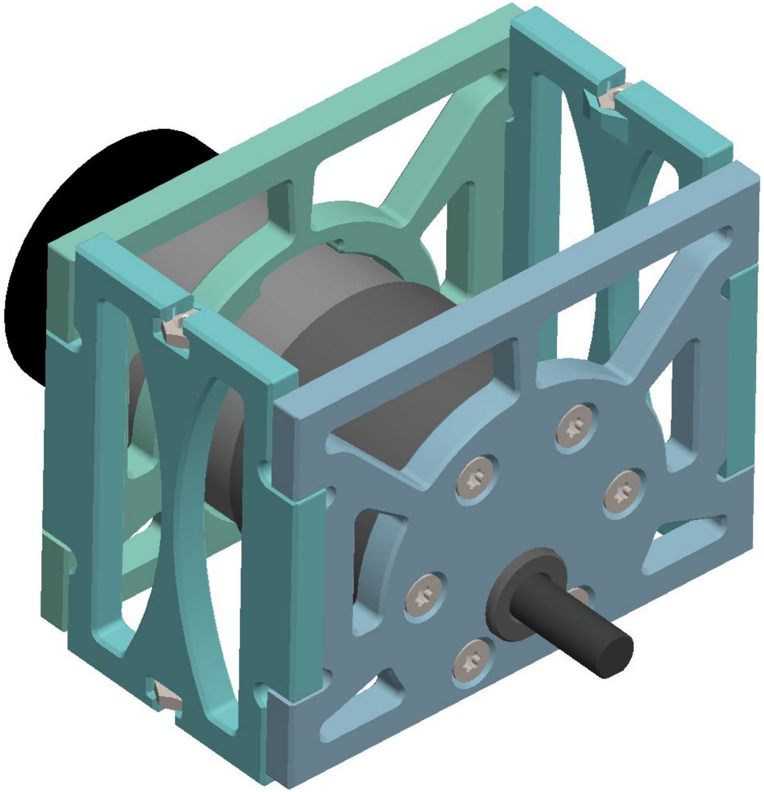

Push wheel coupler onto motor shaft

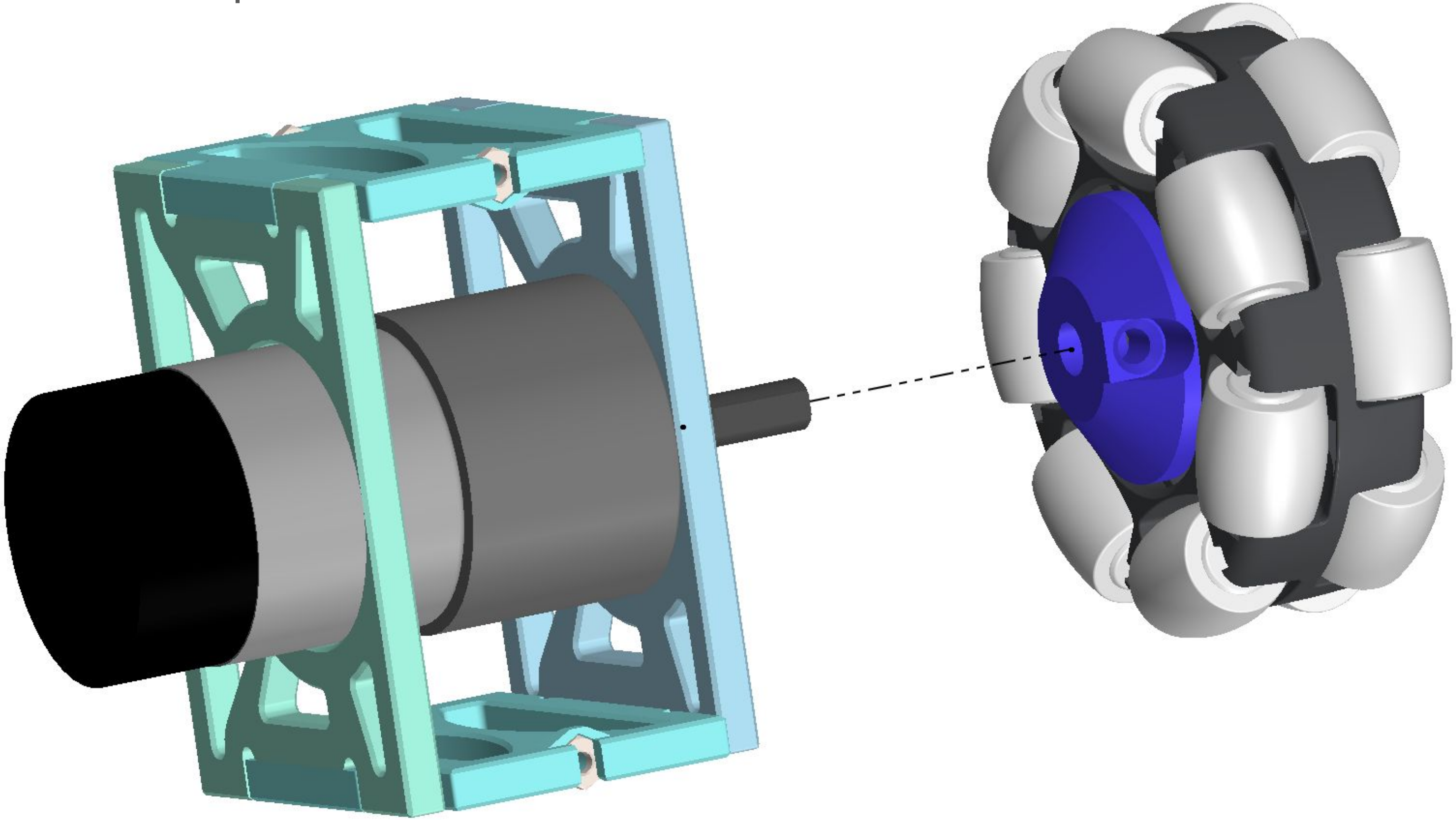

Secure motor to wheel coupler with an M3x8 bolt

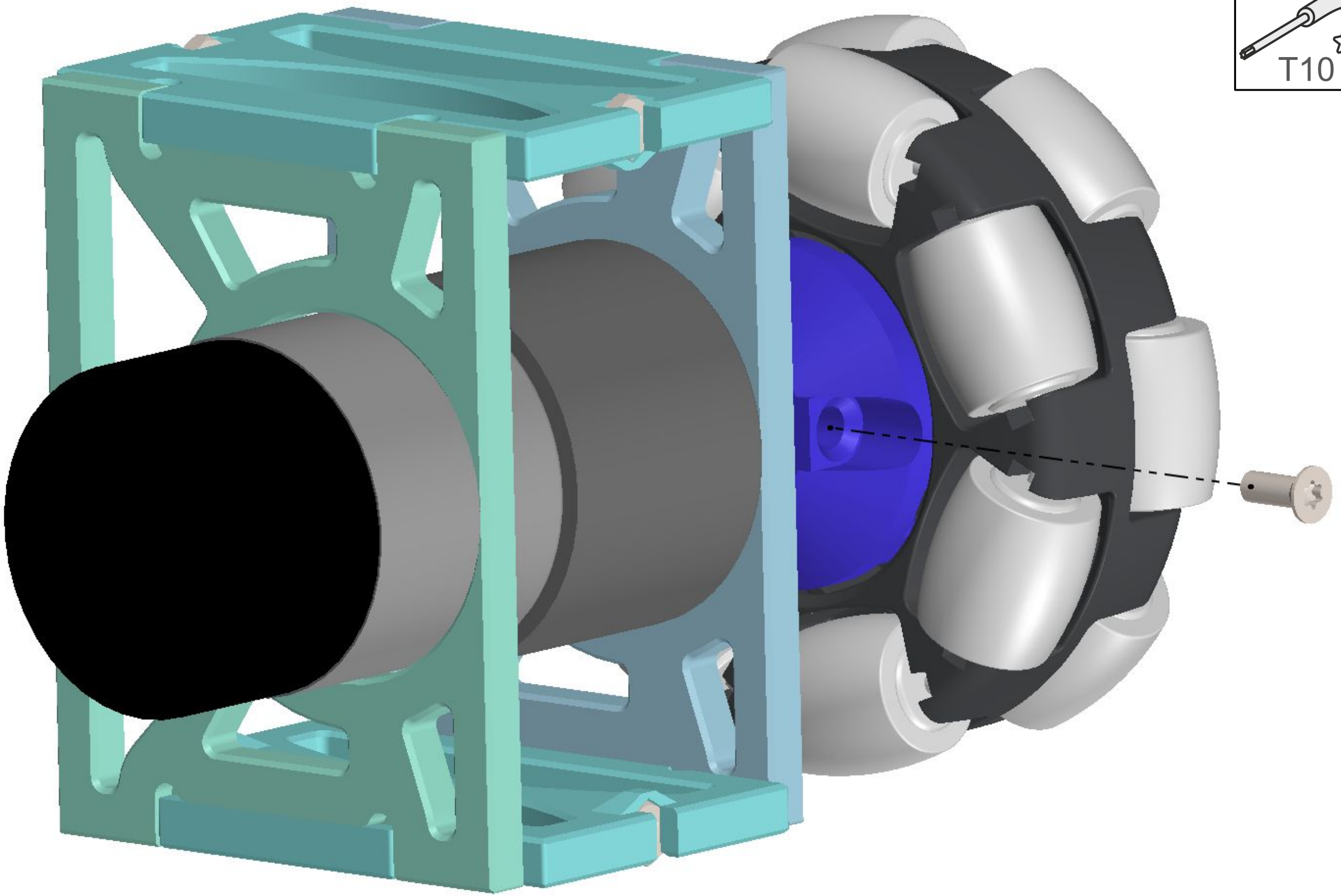

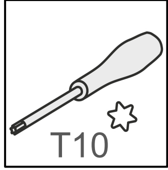

Push motor modules (x3) into frame module along grooves

Avoid shaking to prevent the nuts dislodging

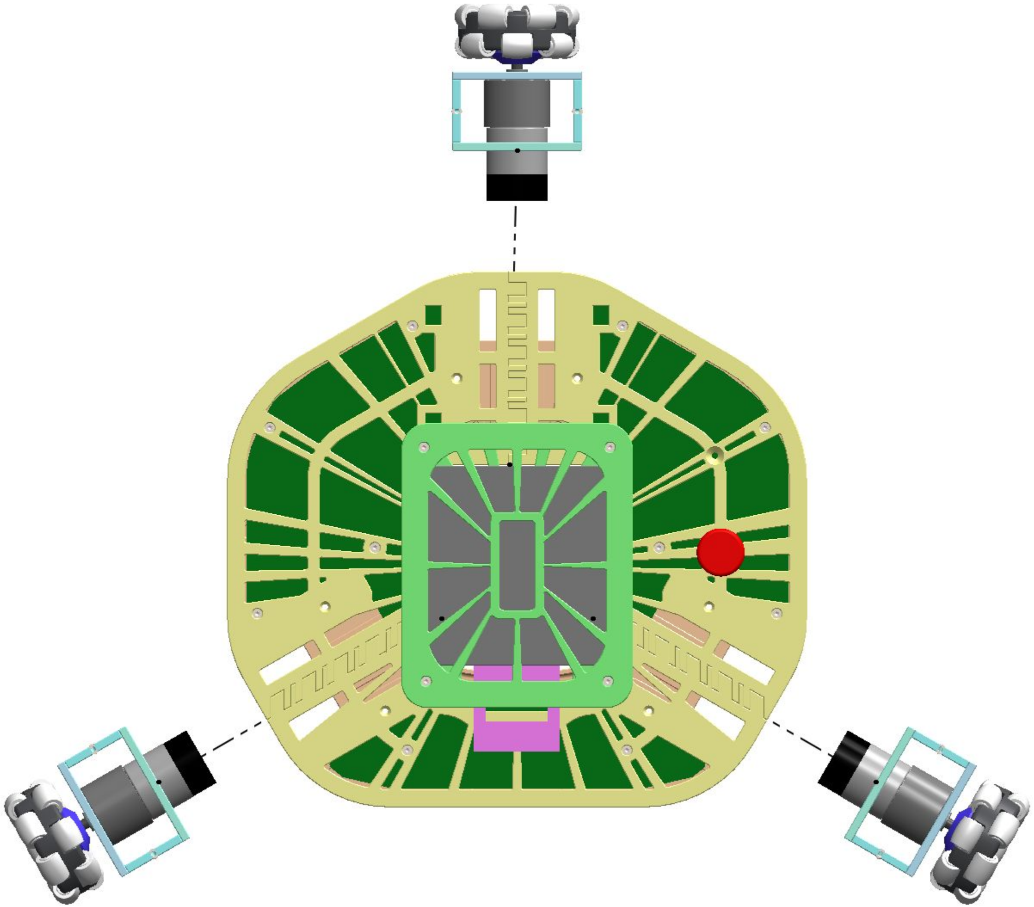

Secure motor modules (x3)
to frame module bottom plate
with M3x8 bolts (x6)

Avoid shaking to avoid
dislodging nuts

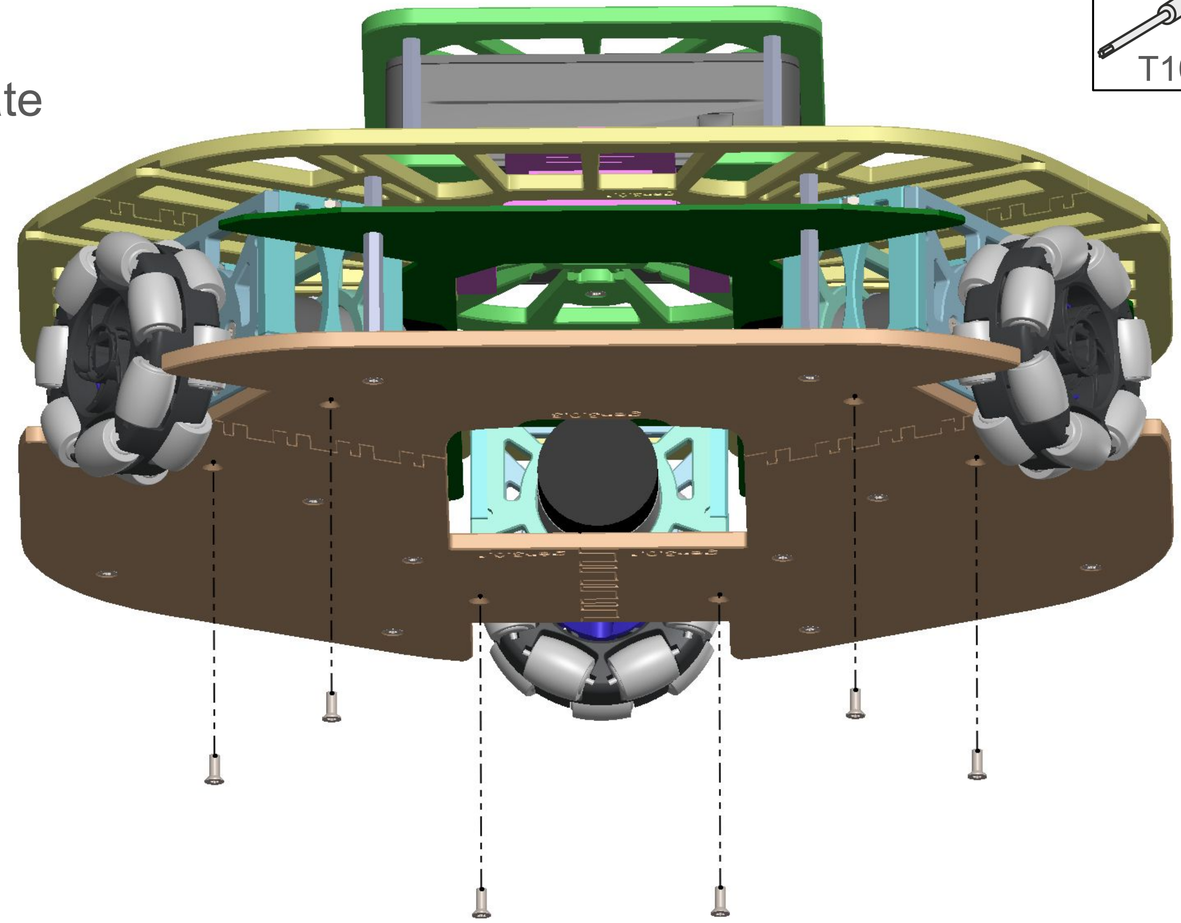

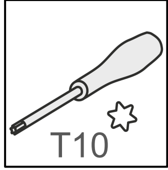

Secure front motor modules (x2) to frame module top plate with M3x8 bolts (x4)

Leave the back motor module unsecured for now

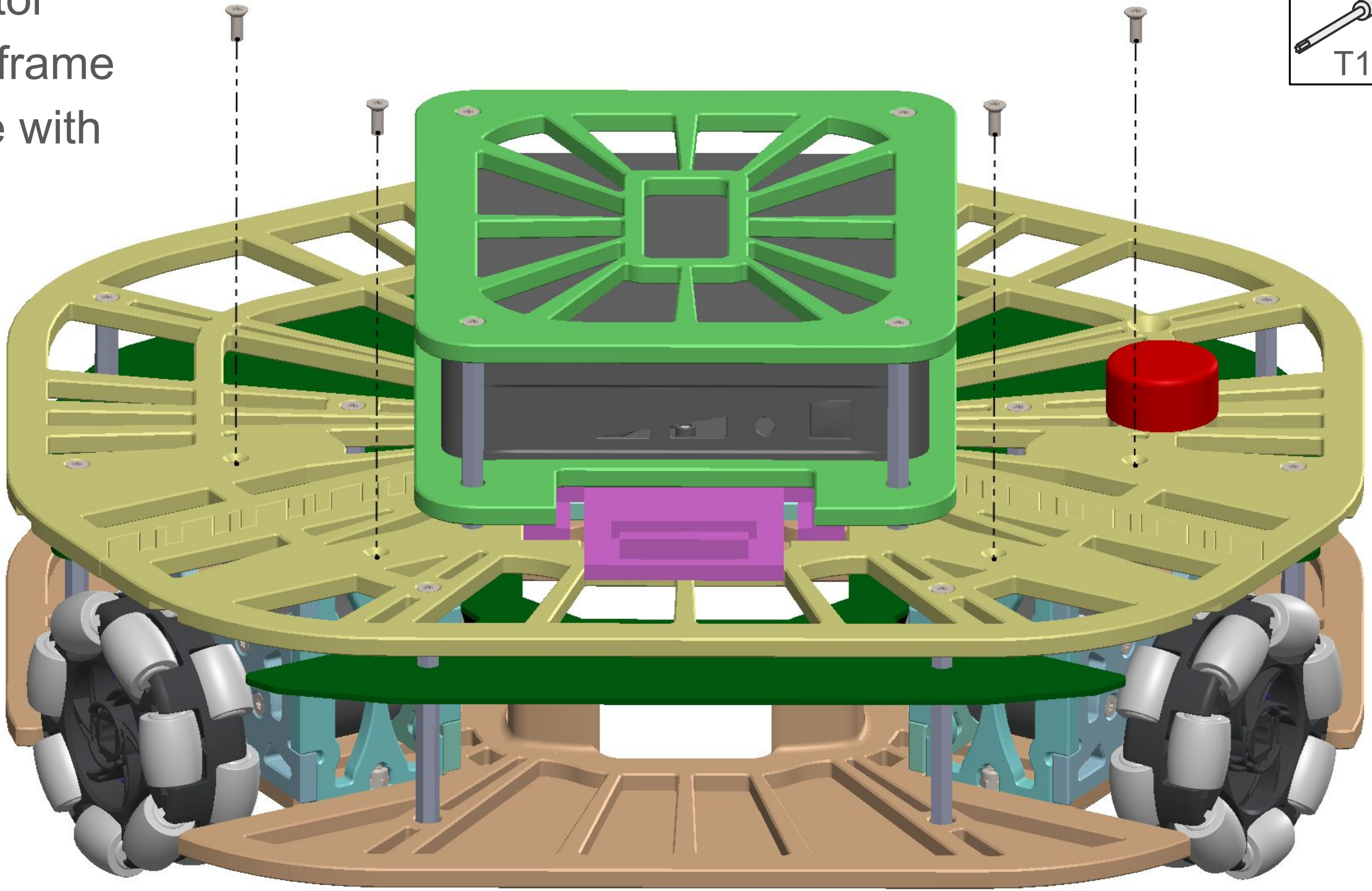

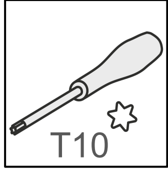

Camera module assembly

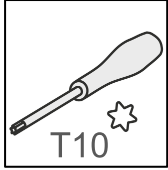

Connect RealSense camera
to camera module centerpiece
with M3x8 bolts (x2)

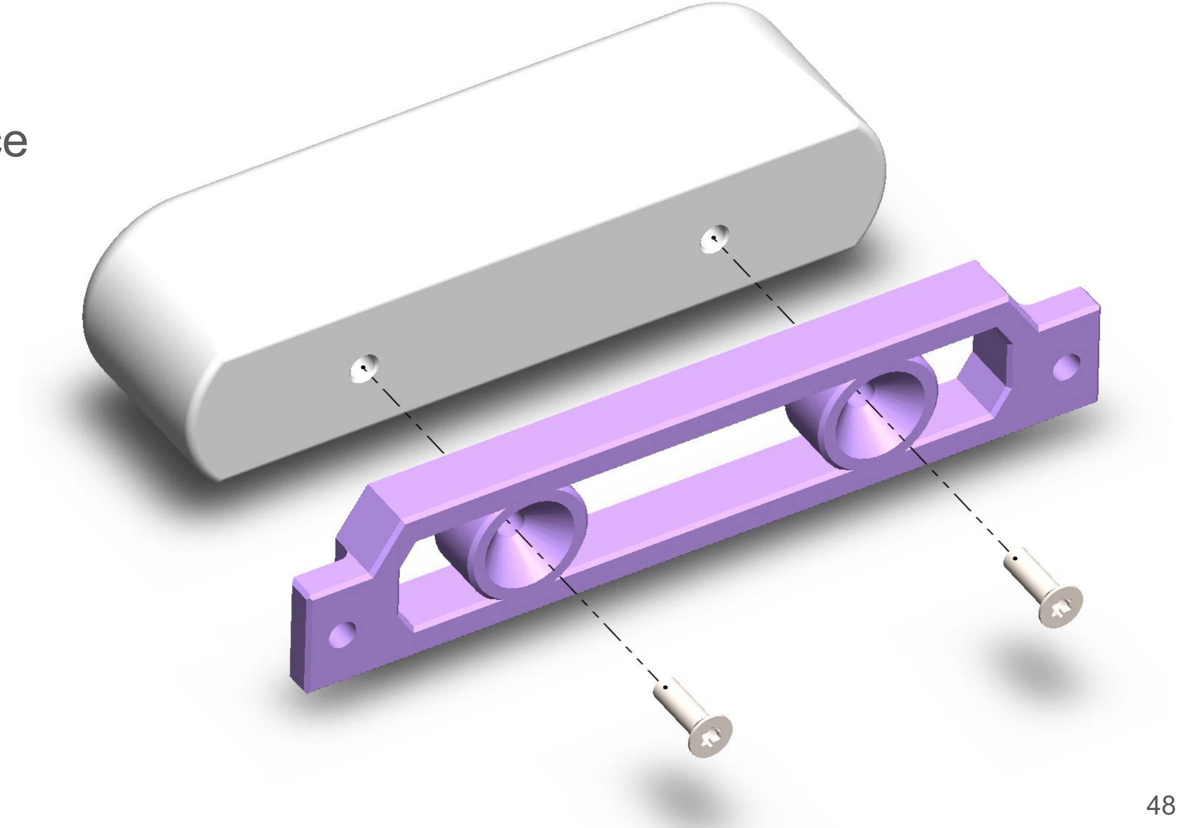

Press camera module sides onto centerpiece ends

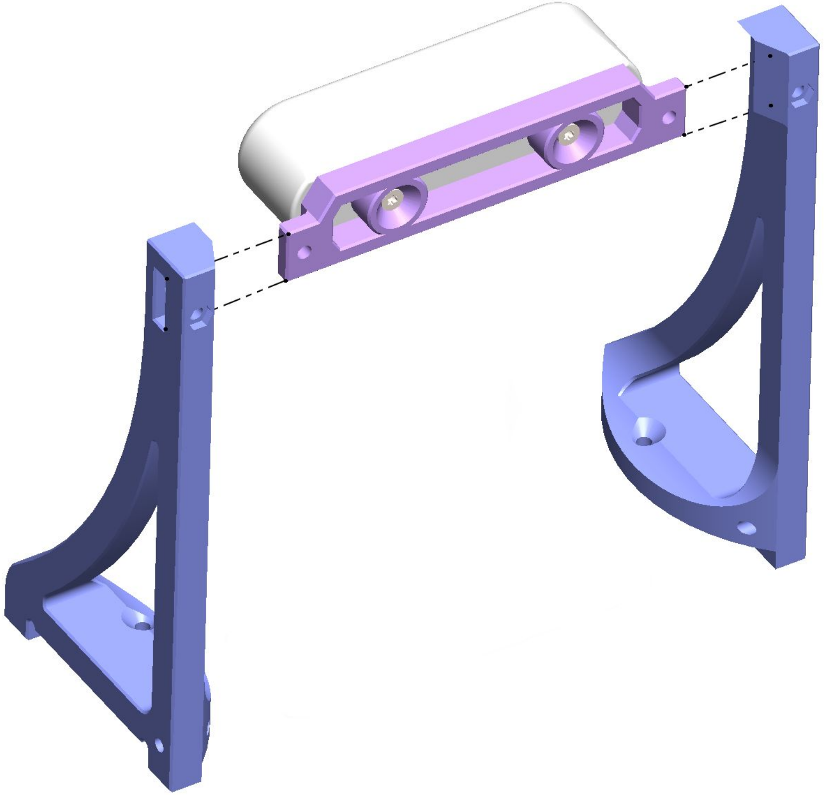

Secure with M3x16 bolts (x2) and M3 nuts (x2)

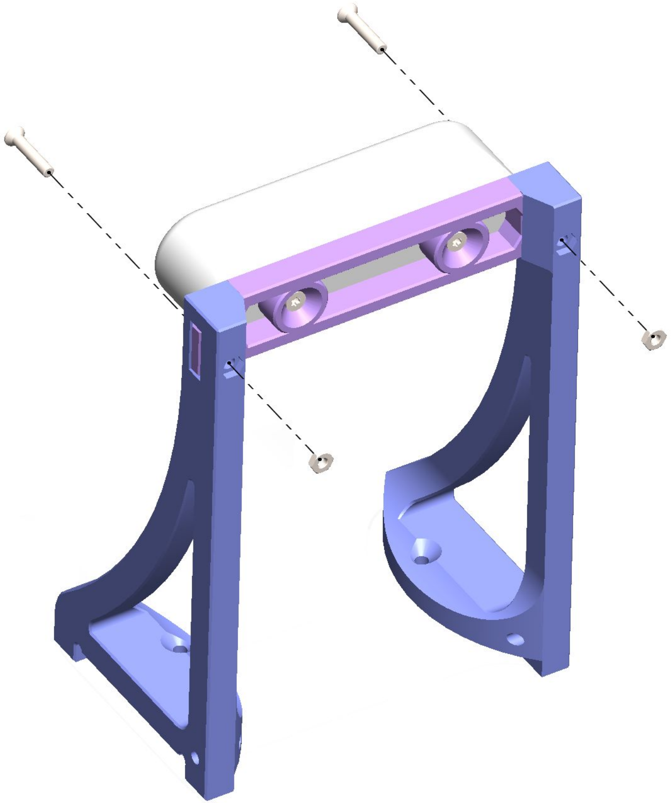

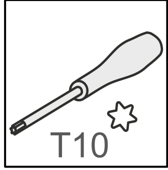

Press camera module into frame module top plate

Secure to back motor module with M3x16 bolts (x2) through holes in frame module top plate

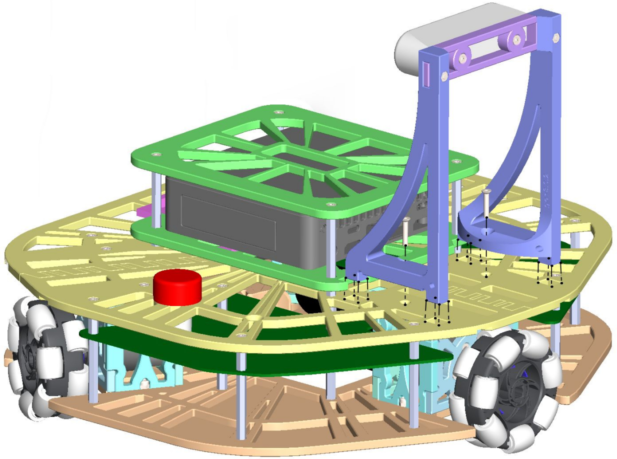

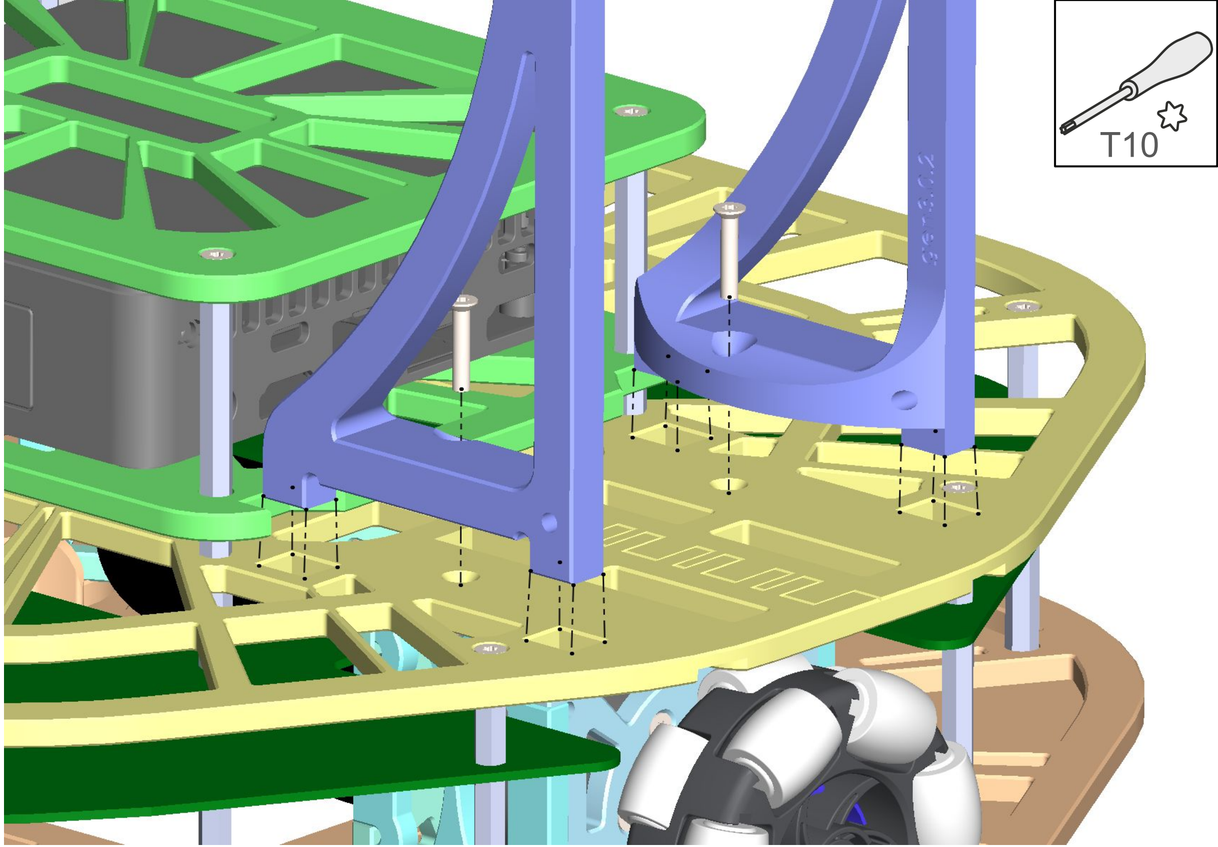

Connect

- (1) power cable
- (2) stop switch
- (3) (4) USB data cables

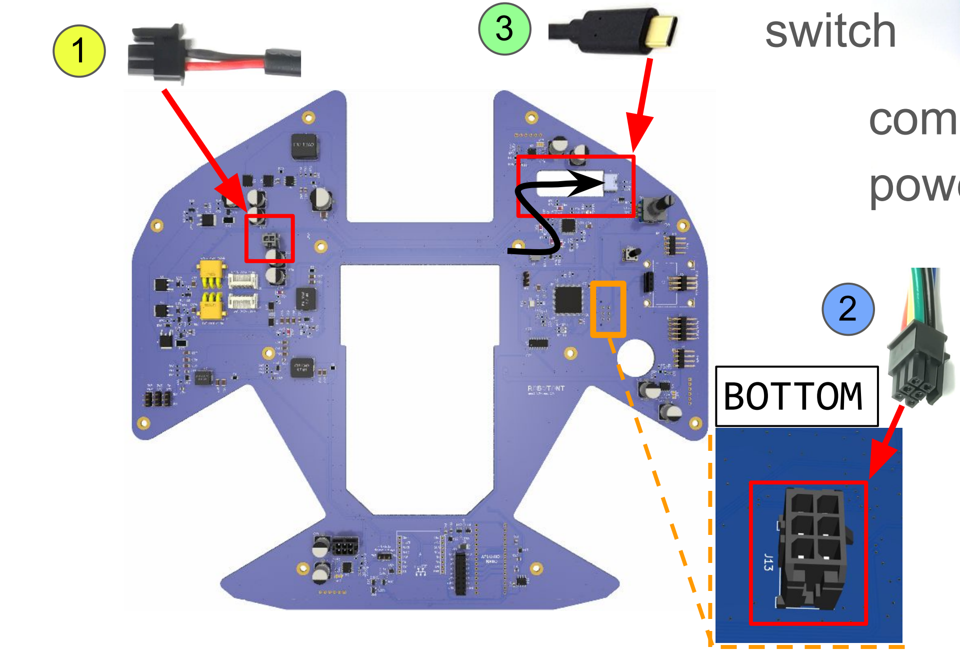

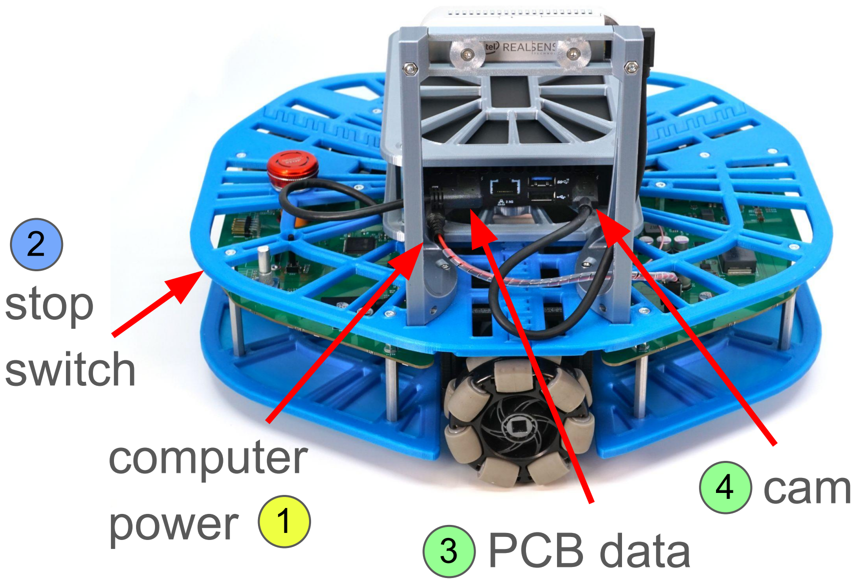

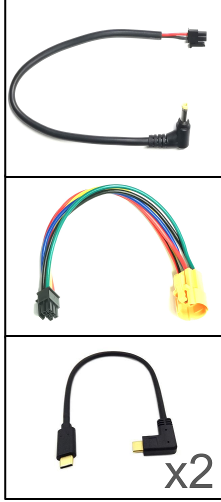

USB connector orientations:

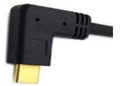		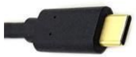
(3) computer	←- - - - →	PCB
(4) camera	←- - - - →	computer

Full assembly

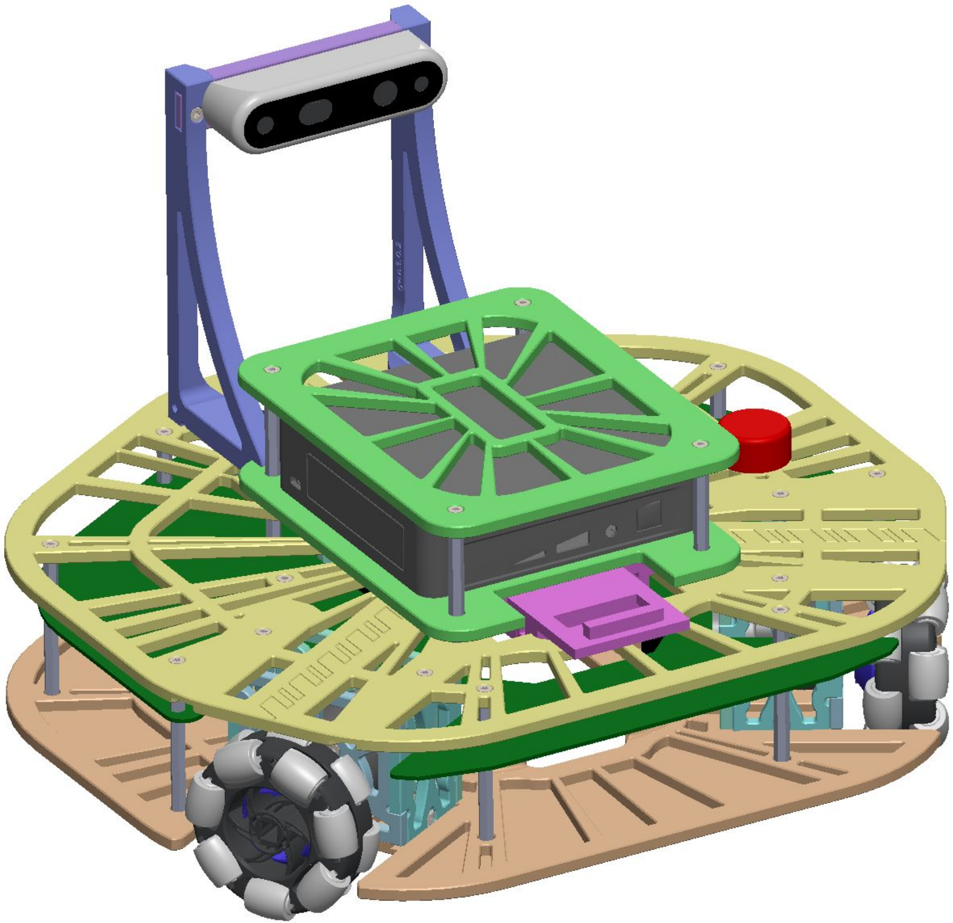

Find latest information about ROBOTONT on GitHub:

https://github.com/robotont

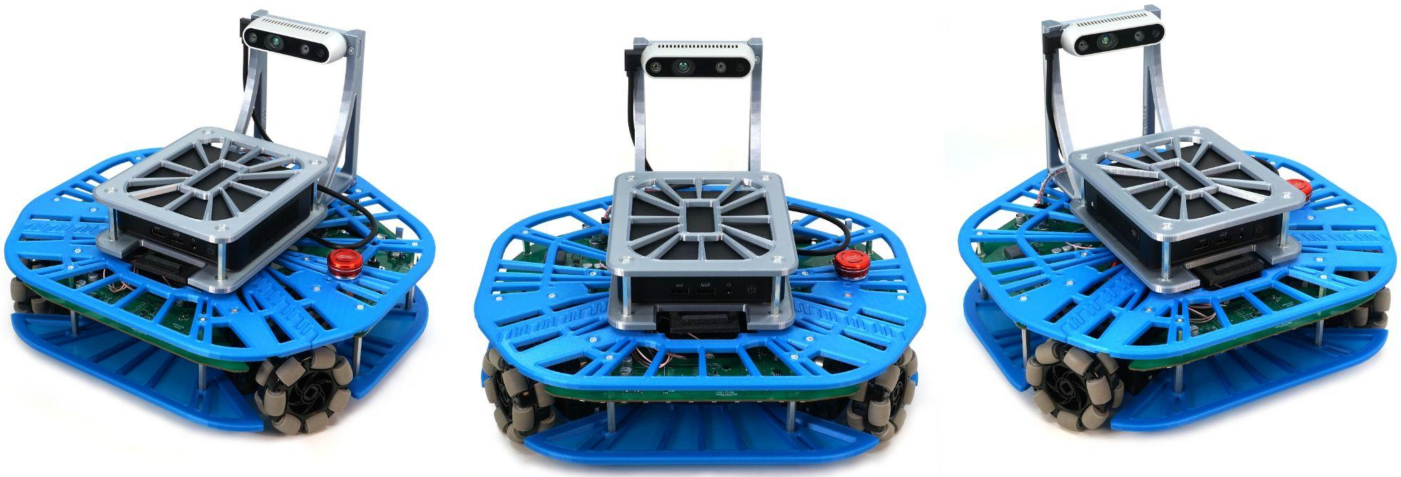

Supplement: Supplementary file 1 [file DataSheet2.pdf]
