# Supplementary material for: Robotont 3–an accessible 3D-printable ROS-supported open-source mobile robot for education and research
Source: Front Robot AI. 2024 Jul 10;11:1406645. doi: 10.3389/frobt.2024.1406645 (PMC11265998; doi:10.3389/frobt.2024.1406645)
Supplement: Supplementary file 2 [file DataSheet1.pdf]

Robotont 3: Bill of Materials

Item	Qty	Cost per piece (€)	Supplier	Product code	Link	Notes
**PREASSEMBLED PCB**						
robotont-electronics-mainboard pcb assembly from jlcpcb	1	120	jlcpcb		jlcpcb.com	Manufacturing files on GitHub: https://github.com/robotont/robotont-electronics-mainboard
**MANUALLY ASSEMBLED TO PCB**						
DC power connector 15A (male) MR30PW-M	2	0.82	tme	MR30PW-M	https://www.tme.eu/en/details/mr30pw-m/dc-power-connectors/amass/	THT connector for battery PWR/GND wires
JST_PH_S6B-PH-K_1x06_P2.00mm_Horizontal	2	0.3	tme	S6B-PH-K-S	https://www.tme.eu/ee/details/s6b-ph-k-s/	connector for battery data pins
Molex_Micro-Fit_3.0_43045-0212_2x01_P3.00mm_Vertical	1	0.8	tme	MX-43045-0212	https://www.tme.eu/ee/details/mx-43045-0212	
Molex_Micro-Fit_3.0_43045-0612_2x03_P3.00mm_Vertical	2	1.1	tme	MX-43045-0612	https://www.tme.eu/ee/details/mx-43045-0612	
PinSocket_1x04_P2.54mm_Vertical	1	0.07	tme	ZL262-4SG	https://www.tme.eu/ee/details/zl262-4sg	For the OLED display
PinSocket_1x15_P2.54mm_Vertical	2	1.1	tme	ZL305-15	https://www.tme.eu/ee/details/zl305-15	For Arduino Nano
PinSocket_2x08_P2.54mm_Vertical	1	0.35	tme	ZL262-16DG	https://www.tme.eu/ee/details/zl262-16dg	
PinHeader_1x03_P2.54mm_Vertical	5	0.02	tme	ZL201-03G	https://www.tme.eu/ee/details/zl201-03g	
PinHeader_1x04_P2.54mm_Horizontal	2	0.04	tme	DS1022-1X4RDF14	https://www.tme.eu/ee/en/details/ds1022-1x4rdf14	
PinHeader_1x06_P2.54mm_Horizontal	3	0.45	tme	826947-6	https://www.tme.eu/ee/en/details/826947-6	
PinHeader_2x03_P2.54mm_Horizontal	1	1.15	tme	826634-3	https://www.tme.eu/ee/details/826634-3	
PinHeader_2x05_P2.54mm_Horizontal	1	1.9	tme	MX-90122-0765	https://www.tme.eu/ee/details/mx-90122-0765	
SOCKET MIKROBUS 16 THT (or 2 x 1x08 PinSocket P2.54)	1	0.9	tme	MIKROE-4247	https://www.tme.eu/ee/en/details/mikroe-4247/development-kits-accessories/mikroe/socket-mikrobus-16-tht/	
RotaryEncoder_Switch	1	0.85	tme	EC11E20-20P20C-SW	https://www.tme.eu/ee/details/ec11e20-20p20c-sw	
SW_PUSH_L6mm_W6mm_H20mm	1	0.1	ali		https://www.aliexpress.com/item/32912263133.html	
SSD1306_0.96_Oled module (NB pin order: GND, VCC, SCL, SDA)	1	3	ali		https://www.aliexpress.com/item/1005001861762328.html	
**CABLING & CONNECTORS**						
Molex_Micro-Fit_3.0_2x01_Female	1	0.7	tme	MX-43025-0200	https://www.tme.eu/ee/details/mx-43025-0200	PCB<->NUC cable (to PCB)
Molex_Micro-Fit_3.0_2x03_Female	2	0.57	tme	MX-43025-0600	https://www.tme.eu/ee/details/mx-43025-0600	EXT PWR + E-Stop connectors
Micro_Fit_Crimp_Terminal	14	0.1	tme	MX-43030-0001/C	https://www.tme.eu/ee/details/mx-43030-0001_c	
5.5 x 2.5 x 11mm DC connector + 20 cm 0.5 mm2 cable	1	2	tme		https://www.tme.eu/ee/details/a21-tt-c075-050bk	PCB<->NUC cable (to NUC)
USB3.1 Type-c male to right angle Type-c male data cable 30cm	2	6	ali		https://www.aliexpress.com/item/4000078385173.html	NUC<->PCB; NUC<->RealSense
JST_PH_S6B-PH-K_1x06_P2.00mm_Female	2	0.08	tme	PHR-6	https://www.tme.eu/ee/details/phr-6/signaaliuhendused-raster-2-00mm/jst/	
Contact; female; phosphor bronze; tinned; 0.05÷0.22mm2; PH	12	0.03	tme	SPH-002T-P0.5S	https://www.tme.eu/ee/details/sph-002t-p0.5s/signaaliuhendused-raster-2-00mm/jst/	
Wire; SiF; stranded; Cu; 1.5mm2; silicone; red; -60÷180°C; 300V,500V	2	0.985	tme	SIF1.50-R	https://www.tme.eu/ee/en/details/sif1.50-r/silicone-cables-single-core/helukabel/23602/	
Wire; SiF; stranded; Cu; 1.5mm2; silicone; black; -60÷180°C; 100m	2	1.138	tme	SIF1.50-B	https://www.tme.eu/ee/en/details/sif1.50-b/silicone-cables-single-core/helukabel/23601/	
Wire; SiF; stranded; Cu; 0.75mm2; silicone; blue; -60÷180°C; 100m	5	0.599	tme	SIF0.75-BL	https://www.tme.eu/ee/en/details/sif0.75-bl/silicone-cables-single-core/helukabel/23403/	
**OTHER COMPONENTS**						
16mm Stop button (two color common positive) + wiring set; Order a version **without** Resistor in the button!	1	9	ali		https://www.aliexpress.com/item/4000395340007.html	
DC Motor 37Dx68L 19:1 12V 530RPM + enkoder CPR 64 -zamiennik Pololu 4751	3	20	ebmia	89926107550	https://www.ebmia.pl/silniki-dc-z-przekladnia/217071-silnik-z-przekladnia-37dx68l-19-1-12v-530rpm-enkoder-cpr-64-zamiennik-pololu-4751.html	
**Onboard computer**						
Barebone Intel NUC13ANKI5	1	462	ordi		https://ordi.eu/ordi-miniarvutid-ordi-5nuc-13gen	
DDR4 16GB 3200MHz NB Kingston	1	37.2	ordi		https://ordi.eu/malu-ddr4-16gb-3200mhz-nb-kingston	
SSD 250GB Kingst. NV2 M.2 NVMe	1	24	ordi		https://ordi.eu/kovakettad-ssd-ssd-250gb-kingst-nv2-m-2-nvme	
**BATTERY AND LOCKING MECHANISM**						
Battery charger	1	75	makserv	195585-0	https://www.makserv.ee/toode/makita-kiirlaadija-dc18rc-72-18-v/	
Battery	2	100	makserv	196673-6	https://www.makserv.ee/toode/bl1850b/	
PCB for locking mechanism	1	8.54	jlcpcb		jlcpcb.com	Manufacturing files on GitHub: https://github.com/robotont/robotont-electronics-battery-adapter
Spring loaded connector	1	2.17	mouser	652-70AAJ-6-M0G	https://www.mouser.ee/ProductDetail/Bourns/70AAJ-6-M0G?qs=nS9uH9%252BmxhxEMX64nt74%252Bw%3D%3D	
JST PH connector right angle male S6B-PH-K-S(LF)(SN)	1	0.054	jlcpcb	C157920	https://jlcpcb.com/partdetail/Jst_SalesAmerica-S6B_PH_K_S_LF_SN/C157920	
DC power connector 15A (female) MR30-FB	2	0.31	tme	MR30-FB	https://www.tme.eu/en/details/mr30-fb/dc-power-connectors/amass/	connector for battery PWR/GND wires
Battery contacts: Sheet metal strip (11x105 mm2)	1		local hardware store			
**CHASSIS**						
Spacer FF M3 x 40 mm	16	0.175	tme	TFF-M3X40/DR123	https://www.tme.eu/ee/details/tff-m3x40_dr123	
Spacer MF M3 x 15 mm	12	0.15	tme	TFM-M3X15/DR223	https://www.tme.eu/ee/details/tfm-m3x15_dr223	
Spacer MF M3 x 6 mm	4	0.14	tme	B3X6/BN3318	https://www.tme.eu/ee/details/b3x6_bn3318	
Bolt Torx-10 Countersunk M3 x 8 mm	65	0.0291	tme	B3X8/BN3803	https://www.tme.eu/ee/details/b3x8_bn3803	
Bolt Torx-10 Countersunk M3 x 16 mm	4	0.0399	tme	B3X16/BN3803	https://www.tme.eu/ee/details/b3x16_bn3803	
Nut M3	14	0.0085	tme	B3/BN117	https://www.tme.eu/ee/details/b3_bn117	
Insert M3 x 5.9 mm	10	0.5	tme	DR11M3	https://www.tme.eu/ee/details/dr11m3/keermestatud-sisestused/dremec/11m3/	
Filament PLA 650 g	0.65	25	prusa		https://www.prusa3d.com/product/prusament-pla-prusa-galaxy-black-1kg/	
Filament PETG 150 g	0.15	25	prusa		https://www.prusa3d.com/product/prusament-petg-matte-black-1kg/	
**WHEELS**						
2.75″ Double-Roller Omni-Directional Wheels (2-pack)	1.5	25	vex	276-1902	https://goprior.com/?product=2-75-omni-directional-wheel-double-roller-2-pack	
